# Dual Checkpoint Aptamer Immunotherapy: Unveiling Tailored Cancer Treatment Targeting CTLA-4 and NKG2A

**DOI:** 10.3390/cancers16051041

**Published:** 2024-03-04

**Authors:** Mohamad Ammar Ayass, Trivendra Tripathi, Natalya Griko, Tutku Okyay, Ramya Ramankutty Nair, Jin Zhang, Kevin Zhu, Kristen Melendez, Victor Pashkov, Lina Abi-Mosleh

**Affiliations:** Ayass Bioscience LLC, 8501 Wade Blvd, Bld 9, Frisco, TX 75034, USA

**Keywords:** aptamer, cancer, CTLA-4, NKG2A, checkpoint, immunotherapy

## Abstract

**Simple Summary:**

Cancer remains a significant health challenge, with persistent annual increases in new cases and mortality rates. Notably, breast, prostate, and uterine cancers are surging. Economically, cancer care bears an overwhelming burden, with costs varying based on cancer phase and type of treatment. Immunotherapy, including T cell immune-checkpoint inhibitors and CAR-directed T lymphocytes, is pivotal in modern oncology, targeting CTLA-4 and PD-1/PD-L1 pathways. Recent research underscores CTLA-4’s diverse role in immune modulation, impacting T cell activation, IDO induction, and T cell proliferation inhibition. Additionally, recognition of the NKG2A axis, interacting with HLA-E on tumor cells, highlights its significance in immune evasion and cancer progression. Current FDA-approved anti-CTLA-4 therapies exhibit promise but face challenges regarding response rates and immune-related adverse events. This study employed computational methods to design aptamers targeting CTLA4 and NKG2A. Aptamer binding specificity was validated through direct and competitive assays using ELISA and flow cytometry. We demonstrated the biological functionality of AYA22T-R2-13, confirming its specific binding to CTLA-4 and NKG2A, and its potential to enhance CD8 T cell and NK cell activity, promoting in vitro tumor cell lysis in human cell lines.

**Abstract:**

Recent strides in immunotherapy have illuminated the crucial role of CTLA-4 and PD-1/PD-L1 pathways in contemporary oncology, presenting both promises and challenges in response rates and adverse effects. This study employs a computational biology tool (in silico approach) to craft aptamers capable of binding to dual receptors, namely, inhibitory CTLA4 and NKG2A, thereby unleashing both T and NK cells and enhancing CD8^+^ T and NK cell functions for tumor cell lysis. Computational analysis highlighted AYA22T-R2-13 with HADDOCK scores of −78.2 ± 10.2 (with CTLA4), −60.0 ± 4.2 (with NKG2A), and −77.5 ± 5.6 (with CD94/NKG2A). Confirmation of aptamer binding to targeted proteins was attained via ELISA and flow cytometry methods. In vitro biological functionality was assessed using lactate dehydrogenase (LDH) cytotoxicity assay. Direct and competitive assays using ELISA and flow cytometry demonstrated the selective binding of AYA22T-R2-13 to CTLA4 and NKG2A proteins, as well as to the cell surface receptors of IL-2-stimulated T cells and NK cells. This binding was inhibited in the presence of competition from CTLA4 or NKG2A proteins. Remarkably, the blockade of CTLA4 or NKG2A by AYA22T-R2-13 augmented human CD8 T cell- and NK cell-mediated tumor cell lysis in vitro. Our findings highlight the precise binding specificity of AYA22T-R2-13 for CTLA4-B7-1/B7-2 (CD80/CD86) or CD94/NKG2A-HLA-E interactions, positioning it as a valuable tool for immune checkpoint blockade aptamer research in murine tumor models. These in vitro studies establish a promising foundation for further enhancing binding capacity and establishing efficacy and safety in animal models. Consequently, our results underscore the potential of AYA22T-R2-13 in cancer immunotherapy, offering high specificity, low toxicity, and the potential for cost-effective production.

## 1. Introduction

Cancer ranks as the second leading cause of mortality in the United States, constituting a significant public health challenge. With an estimated annual toll of 609,820 deaths attributed to cancer and an anticipated 1.9 million new cases in 2023 [1], its impact remains profound. Among the primary contributors to adult mortality are lung/bronchus, colon and rectum, pancreas, breast, prostate, and liver/intrahepatic bile duct cancer [2]. While a notable 32% decline in cancer-related mortality between 1999 and 2019 has been achieved due to heightened awareness, early diagnosis, preventive measures, combination therapies, and chemotherapy [3], the incidence rates of breast, prostate, and uterine corpus cancers are ascending [1]. The economic burden of cancer care surpasses USD 21 billion annually in the United States, as reported by the National Cancer Institute in 2019, with per-patient average costs for medical services ranging from USD 5518 to USD 109,727 and oral prescription drug costs ranging from USD 1041 to USD 4372, contingent upon the phase and site of cancer [4,5]. Immunotherapy has emerged as a cornerstone in modern oncology. Significant clinical responses have been elicited with T cell immune-checkpoint inhibitors and chimeric antigen receptor-directed (CAR-directed) T lymphocytes [6]. Crucially, the cytotoxic T-lymphocyte–associated antigen 4 (CTLA-4)/B7 and programmed death 1 (PD-1)/programmed cell death-ligand 1 (PD-L1) pathways represent pivotal immune checkpoints regulating T cell immune function at different activation phases [7]. Recent research delineates CTLA-4’s multifaceted role in immune modulation, encompassing inhibition of T cell activation, induction of indoleamine 2,3-dioxygenase (IDO) via reverse signaling with B7, and suppression of T cell proliferation, among other functions [7]. Furthermore, persistent CTLA-4 expression has been linked to susceptibility to various solid tumors, with differing expression levels potentially influencing cancer progression and clinical outcomes [8,9,10,11]. Of late, the NKG2A axis, a novel immune checkpoint interacting with HLA-E on tumor cells, has been identified. Its interaction suppresses the cytolytic function of natural killer (NK) cells and CD8^+^ T-cells in the tumor microenvironment, contributing to immune evasion and cancer progression [12,13]. Presently, only two anti-CTLA-4 human monoclonal antibodies (Ipilimumab and Tremelimumab) are FDA-approved for use, either alone or in combination therapies, enhancing anti-tumor effects and prognosis [14,15]. However, the response rate to these immunotherapies remains limited, and immune-related adverse events (irAEs) pose considerable risks [15,16]. IrAEs can range from mild (such as diarrhea, nausea, skin rash, and others) to more severe (such as colitis, hepatitis, inflammation of the lung, pneumonitis, kidney failure, myocarditis, or inflammation of the heart). Also, immune checkpoint inhibitors may cause the immune system to attack normal organs and tissues in any part of the body and impair their function [17,18]. Therefore, the demand for an effective and safe anti-CTLA-4 inhibitor is unmet and further evaluation of other immunotherapeutic approaches is necessary. Aptamers are short synthetic single-stranded nucleic acids or peptides that bind to target molecules with high specificity. The first anti-CTLA-4 2F’-RNA aptamer was reported by Gilboa’s group and they showed that this aptamer could bind CTLA-4 specifically and inhibit CTLA-4 function in vitro and enhance tumor immunity in mice [19]. Also, an anti-CTLA-4 DNA aptamer [20] and a dual function aptamer (anti-CTLA-4/PD-L1) [21] have shown that an anti-CLTA-4 aptamer can promote T lymphocyte proliferation, inhibit tumor growth, and strengthen anti-tumor immunity. As described above, aptamers hold great promise for utilization in immunotherapy due to their ability to modulate the immune response by binding to and inhibiting specific immune cells or proteins, or by enhancing the function of certain immune cells.

In this study, by using a computational biology tool (silico approach), we developed AYA22T-R2-13, a CTLA4/NKG2A dual receptor aptamer that blocks the inhibitory CTLA4/NKG2A receptors unleashing both T and NK cells and promotes NK and CD8^+^ T cell effector functions of tumor cell lysis. These findings underscore the promise of AYA22T-R2-13 in cancer immunotherapy, offering high specificity, low toxicity, and the potential for cost-effective production.

## 2. Materials and Methods

### 2.1. Initial Aptamer Generation

Fifty aptamer sequences, each consisting of 41 nucleotides, were produced using a random sequence generator to establish a diverse population of candidates for further analysis. The nucleotide composition of these sequences maintained an equal ratio of 1:1:1:1 of adenine (A), uracil (U), guanine (G), and cytosine (C). This ratio is crucial as it reduces bias towards specific nucleotide motifs and shows a balanced representation of the sequence space.

### 2.2. Iterative Selection and Mutation

An iterative selection process was then carried out to refine and improve the aptamer sequences. The top 15 aptamers with the lowest docking scores, as determined by structure prediction and docking simulation, were selected for the next round of evolution. To introduce diversity into the aptamer pool, two sites within each selected aptamer sequence were randomly chosen, and a random nucleotide mutation was introduced at each site. The mutation involved replacing the original nucleotide with another nucleotide (A, U, G, or C) with equal probability. Forty out of the 50 new aptamers were generated based on the sequences from the previous round, ensuring a degree of continuity and cumulative improvement. The remaining 10 aptamers were randomly generated following the same protocol as in the initial generation step. This process was repeated for a total of 10 rounds of selection. This iterative approach aimed to gradually optimize the aptamer sequences for enhanced binding affinity and specificity towards the target CTLA4 and NKG2A protein. After the 10th round of iterative selection, the top nine aptamers were identified based on their docking scores and RMSD scores. These aptamers exhibited the most favorable binding interactions with the target CTLA4 and NKG2A proteins. The selection of these top aptamers marked the completion of the computational phase and paved the way for subsequent experimental validation studies.

### 2.3. Secondary and Tertiary Structure Prediction

At each round of selection, a comprehensive computational predication approach was utilized to predict the secondary and tertiary structures of the generated aptamers. The DNA fold service [22] is a well-established bioinformatic tool that uses advanced algorithms and thermodynamic calculations to predict secondary structures, and calculates the energy associated with different potential folding patterns for each aptamer sequence. The sequences with the lowest energy were selected for further investigation. To determine the three-dimensional (3D) structure of the selected aptamer sequences, we first utilized the RNAcomposer tool [23,24], which is known for its accuracy and reliability in predicting RNA 3D structures. Each aptamer sequence, paired with its corresponding secondary structure, was provided as input to RNAcomposer. Energy minimization procedures were then applied to refine and optimize the 3D structures of the aptamers, enhancing their stability and accuracy.

### 2.4. Oligonucleotides

The prediction of the aptamer sequences initially utilized uracil during computational design to represent RNA-like structures. However, deoxyuridine was substituted when ordering the physical DNA aptamer strands for experimental validation and binding assays. Though deoxyuridine and uracil share the uracil nucleobase structure, deoxyuridine also contains a deoxyribose sugar bound to this structure. When incorporated into the DNA aptamers in place of uracil, deoxyuridine forms additional stabilizing interactions, like N-glycosidic bonds and favorable base stacking, without altering the computationally designed folding or binding sites. This reinforces the DNA aptamer structure and resilience. The resulting DNA aptamers with deoxyuridine maintain the predicted structural stability and function to a greater degree than if using uracil. So while the predicted sequences successfully guided aptamer design, deoxyuridine enhances the real DNA aptamers’ stability, integrity, and binding capability for experimental use [25]. Aptamer libraries and modified aptamers were synthesized by IDT (Integrated DNA Technologies, Coralville, IA, USA). The sequences for dual checkpoints, CTLA-4/NKG2A aptamers (AYA22T-aptamers) are listed in the Supplementary Materials, Table S1. All the aptamers were activated prior to the experiments in 2 mL of activation buffer (20 mM Na-phosphate, 0.2 M NaCl and 0.5 mM MgCl_2_, pH 7.4) at 95 °C for 10 min followed by 20 min incubation on ice [26].

### 2.5. Docking Simulation

To investigate the potential interactions between the aptamers and CTLA4 and NKG2A proteins, docking simulations were performed using HADDOCK 2.4 [27,28]. The predicted CTLA4 3D structure was obtained from the RCSB protein data bank [29] with PDB ID: 1I8L. NKG2A and the NKG2A/CD94 heterodimer were obtained from the RCSB protein data bank [29] with PDB ID:3BDW. PyMOL (The PyMOL Molecular Graphics System, Version 2.0, Schrödinger, LLC., New York, NY, USA) was used to visualize the proteins and prep them for docking. The aptamer structures generated during the iterative selection were docked onto these epitopes using appropriate software and algorithms. Docking scores were recorded as a measure of the binding affinity between each aptamer and the target CTLA4 and NKG2A proteins.

### 2.6. Cell Lines

The human cancer cell lines, namely HCT-15 (CCL-225), COLO-205 (CCL-222), LNCaP clone FGC (CRL1740), BT-549 (HTB-122), A-375 (CRL-1619), A-549 (CCL-185), and BT-474 (HTB-20), were procured from the American Type Culture Collection (ATCC, Manassas, VA, USA). These cell lines were maintained according to ATCC handling guidelines. HCT-15, COLO-205, LNCaP clone FGC, BT-549 cells, and human peripheral blood mononuclear cells (hPBMCs) were cultured in RPMI-1640 medium (ATCC, Manassas, VA, USA). A-375 cells were cultured in Dulbecco’s Modified Eagle’s medium (DMEM) (Gibco, Grand Island, NY, USA), while A-549 cells were cultured in F-12K medium (ATCC, Manassas, VA, USA). The BT-474 cell line was cultured in Hybri-Care medium (ATCC, Manassas, VA, USA) supplemented with 1.5 g/L sodium bicarbonate (Sigma-Aldrich, St. Louis, MO, USA). All culture media were supplemented with 10% fetal bovine serum (FBS) (HyClone, South Logan, UT, USA), Pen Strep (Gibco, Grand Island, NY, USA), and MEM NEAA (Gibco, Grand Island, NY, USA). Additionally, HEPES (Gibco, Grand Island, NY, USA), and sodium pyruvate (Gibco, Grand Island, NY, USA) were supplemented when not provided commercially in the media. Culturing of all cell lines was carried out at 37 °C in a humidified 5% CO_2_ environment, adhering to standard cell culture protocols. Materials and key resources used in the methods are mentioned in detail in Supplementary Materials, Table S2.

### 2.7. Competitive Inhibition by ELISA

An ELISA-based competition assay was conducted to determine the specificity of binding for the AYA22T-aptamers (AYA22T-R2-13, AYA22T-R3-25, AYA22T-R2-81, AYA22T-R3-20, AYA22T-R3-56, and AYA22T-R2-63) to CTLA4/NKG2A proteins. A MaxiSorp plate was coated overnight with (5.0 µg/mL of human CTLA-4/CD152 or human NKG2A/CD159a proteins (Acro Biosystems, Newark, DE, USA) prepared in a 50 mM carbonate–bicarbonate solution. Subsequently, the plate was blocked for 1 h with 2% BSA in 1 × PBS/Tween, supplemented with 1 mM MgCl_2_. After extensive washing with PBS, pH 7.4, biotinylated AYA22- aptamers at a constant concentration of 5.0 µM were introduced to the presence of varying concentrations of CTLA4, PD-1, or NKG2A proteins, respectively. The plate was incubated at room temperature for 1 h. The binding of the aptamer was detected using streptavidin-HRP (Thermo fisher Scientific, Plaquemine, LA, USA) in combination with TMB. The reaction was halted by the addition of 1 M H_2_SO_4_, and subsequent color changes were assessed using a spectrophotometer at 450 nm. During the ELISA process, the plate underwent meticulous washing six times with a wash buffer using a plate shaker set at 900 rpm for 5 min after each procedural step, ensuring comprehensive removal of unbound substances and maintaining assay accuracy. The absorbance of each well was measured at 450 nm using an automatic microplate reader. Percent inhibition was calculated as described in [30]: percent inhibition = [1 − (A_inhibitor_/A_uninhibitor_)] × 100.

### 2.8. Molar to Molar Competition Assay by ELISA

The method for analyzing the binding of aptamers to human CTLA-4/CD152 and human NKG2A/CD159a proteins through ELISA was comprised of precise steps. The protocol is described in detail in the Supplementary Material.

### 2.9. ELISA-Based Binding Assay of AYA22T-R2-13 with CTLA-4/CD152 Protein

Initially, a MaxiSorp plate was coated overnight with 5 µg/mL of human CTLA-4/CD152 (Acro Biosystems, Newark, DE, USA) prepared in a 50 mM carbonate–bicarbonate solution. Concurrently, the plate was also coated with BSA and D-dimer proteins at a concentration of 5 µg/mL to serve as controls. The subsequent day, the plate underwent blocking for 1 h using 2% BSA in 1 mM MgCl_2_ supplemented 1× PBS/Tween solution. Biotinylated AYA22T-R2-13 at varying concentrations was then added to the wells in duplicate and incubated at room temperature for 1 h. After extensive washing steps, HRP-conjugated streptavidin (Thermo fisher Scientific, Plaquemine, LA, USA) was utilized in conjunction with TMB for colorimetric detection. The reaction was terminated by the addition of 1 M H_2_SO_2_, following which the color change was measured using a spectrophotometer at 450 nm.

### 2.10. Flow Cytometry-Based Binding Assay of AYA22T-Aptamers to CTLA4 Protein

The investigation of biotinylated aptamer binding to human CTLA-4/CD152 protein (Acro Biosystems, Newark, DE, USA) via streptavidin-coated fluorescent particles kit was conducted using a meticulous flow cytometry-based approach [30]. The procedures used for flow cytometry-based binding assay are described in the Supplementary Material.

### 2.11. PBMCs Isolation

The peripheral blood mononuclear cells (PBMCs) were isolated from healthy donors’ buffy coats obtained from Carter BloodCare using a Ficoll–Paque Plus density-gradient centrifugation method (Cytiva Life Sciences, Burlington, NJ, USA).The protocol is described in detail in the Supplementary Material.

### 2.12. Isolation and Stimulation of CD8^+^ T Cells and NK Cells

The procedures used for isolation and stimulation of CD8+ T cells and NK cells are described in the Supplementary Material.

### 2.13. Surface Staining of Tumor Cells

The analysis of IFN-γ-stimulated tumor cells for the expression of surface markers, including CD80, CD86, PDL-1, and HLA-E, along with their respective isotype control antibodies, was performed utilizing flow cytometry. The protocol is described in detail in the Supplementary Material.

### 2.14. Surface Staining of Immune Cells including Enriched CD8 T Cells, Total T Cells and NK Cells

The assessment of CTLA-4 expression on various immune cell subsets and NKG2A expression on NK cells was conducted through surface staining methodologies. The protocol is described in detail in the Supplementary Material.

### 2.15. Competitive Binding of AYA22T-R2-13 to CTLA4/NKG2A Surface Receptors on CD8 T Cells

The evaluation of AYA22T-R2-13 binding to CTLA-4/NKG2A receptors expressed on activated CD8 T cells was performed using surface staining techniques. The protocol is described in detail in the Supplementary Material.

### 2.16. Detection of Cell Surface CTLA4 Binding to AYA22T-R2-13 via Competitive Binding Inhibition Assay

The assessment of AYA22T-R2-13 binding to the native cell surface CTLA-4 receptor, expressed on activated CD8 T cells, was conducted through a competitive inhibition binding assay. The protocol is described in detail in the Supplementary Material.

### 2.17. Sulforhodamine B (SRB) Cell Cytotoxicity Assay

Cell viability subsequent to aptamer exposure was assessed using SRB cell cytotoxicity colorimetric assays [31,32]. The SRB assay is described in detail in the Supplementary Material.

### 2.18. CD8^+^ T-Cell-Mediated Tumor Cell Killing Assay

The CD8^+^ T-cell-mediated tumor cell killing [33] assay was conducted following a series of controlled steps. The protocol is described in detail in the Supplementary Material.

### 2.19. NK Cell-Mediated Tumor Cell Killing Assay

The NK cell-mediated tumor cell-killing assay involved a methodical series of procedures. The protocol is described in detail in the Supplementary Material.

### 2.20. Killing Assay in the Presence of Human Serum

The protocol is described in detail in the Supplementary Material.

### 2.21. Lactate Dehydrogenase (LDH) Assay

Cytotoxicity was assessed by lactate dehydrogenase (LDH) release from cancer cells into the culture medium [19,34]. The protocol is described in the Supplementary Material.

### 2.22. Surface and Intra-Cellular Staining

Initially, the co-culture plate was subjected to centrifugation at 500× *g* for 5 min, resulting in the collection of the supernatant for LDH assay analysis. Subsequently, to prevent nonspecific binding, Fc receptors of the cells were blocked using Anti-Hu Fc Receptor Binding Inhibitor (Invitrogen, Carlsbad, CA, USA) at a 1:50 dilution. Following this, the cells were stained with fixable viability dye for 15 min at 4 °C. The cells were then thoroughly washed with 1× PBS (without CaCl_2_ & MgCl_2_) and stained with Alexa Fluor700-labeled anti-human CD3 surface marker, followed by incubation at 4 ° for 30 min. Upon completion of the surface staining, the cells underwent fixation and permeabilization using BD Cytofix/Cytoperm solution (BD Biosciences, San Diego, CA, USA) for 20 min at 4 °C. Afterward, the cells were washed with 1× Perm/Wash buffer (BD Biosciences, San Diego, CA, USA) and subsequently stained with granzyme-B and perforin at a dilution of 1:50 for 30 min at 4 °C. Subsequent to antibody incubation, excess markers were removed by washing with 1× perm/wash buffer. Finally, 200 µL of 1× PBS (without CaCl_2_ & MgCl_2_) was added to the wells in preparation for subsequent flow cytometry analysis.

## 3. Statistical Analysis

Significance was determined in Prism 9.4.0 (GraphPad Software) using a non-parametric (Mann–Whitney) test or unpaired Student’s *t* test for two-group comparisons. Data are expressed as mean ± SD. *p* values ≤ 0.05 were considered statistically significant. * denotes *p* ≤ 0.05, ** denotes *p* ≤ 0.01, *** denotes *p* ≤ 0.001, and **** denotes *p* < 0.0001.

## 4. Results

### 4.1. Design and Molecular Docking Analysis of Aptamers Targeting CTLA4 and NKG2A Proteins Using In Silico Approaches

**Figure 1:**
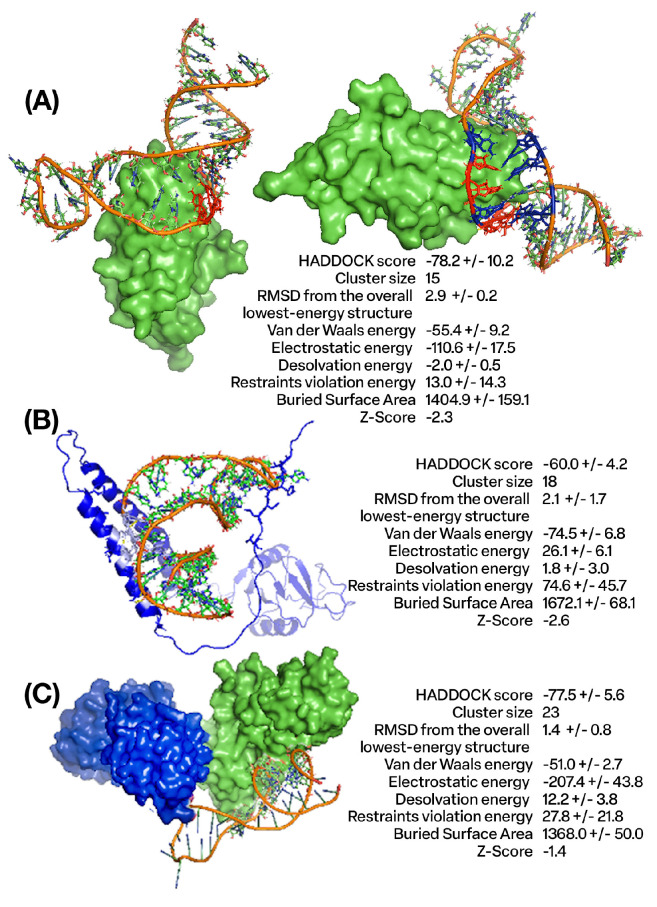
In silico binding of AYA22T-R2-13 with CTLA4 and NKG2A proteins. (**A**) Depicts the docking complex of CTLA4 protein and AYA22T-R2-13 obtained from HADDOCK docking software [28] and provides a side-oriented view of the docking complex between CTLA4 protein and AYA22T-R2-13 with the HADDOCK output results for the docking analysis of AYA22T-R2-13 to CTLA4 protein. (**B**) Shows the docking complex of NKG2A protein and AYA22T-R2-13 with its HADDOCK output results for the docking analysis of AYA22T-R2-13 to the CD94/NKG2A heterodimer protein complex. (**C**) Illustrates the docking complex of CD94/NKG2A and AYA22T-R2-13 with the HADDOCK output results for the docking analysis of AYA22T-R2-13 to the CD94/NKG2A heterodimer protein complex obtained from HADDOCK docking software.

We utilized an in silico (computational) methodology to rationally design DNA aptamers—short single-stranded oligonucleotides—with specific binding affinity towards the CTLA4 and NKG2A proteins. From an initial pool of nine candidates (Supplementary Materials, Table S1), six aptamers exhibiting the most favorable calculated binding energies were selected for further analysis. Figure 1A visualizes the molecular docking complexes and interactions between the CTLA4 protein and the AYA22T-R2-13 aptamer, generated using the HADDOCK protein–DNA docking software suite (versions 2.4/2.6) [27,28]. These figures provide detailed, side-view representations of the structural complex formed between CTLA4 and AYA22T-R2-13. In contrast, Figure 1B,C illustrate analogous docking analysis outcomes between the AYA22T-R2-13 aptamer and the NKG2A protein, including the CD94/NKG2A heterodimer. Specifically, these figures demonstrate the predicted binding modes and molecular interfaces between NKG2A, CD94/NKG2A, and AYA22T-R2-13, also obtained via HADDOCK. Overall, these computational docking results predict and characterize successful in silico binding between the engineered aptamers and their target proteins. Lower (negative) docking scores typically correlate with stronger theoretical binding affinities and formation of more structurally and energetically favorable complexes, providing a quantitative assessment. The comprehensive structural analysis and biophysical characterization of these docking poses and interfaces (Figure 1) offers valuable mechanistic insights into potential binding modes, molecular recognition, and key contacts governing aptamer/protein interactions. This computational framework establishes a foundation for subsequent empirical binding validation and further optimization of these aptamers. For example, having validated binding and determined favorable aptamer/protein poses, directed evolution and mutagenesis studies can now rationally modify the aptamer sequence to improve affinity and specificity. Specific aptamer nucleotides making key contacts at the binding interface can be mutated in silico and re-docked to evaluate changes in binding score and mode. Additionally, expanding the diversity of the aptamer pool (Supplementary Materials, Table S1) with rational mutations predicted to enhance intermolecular contacts would enable improved candidates to be identified via new rounds of computational docking. Thus, CTLA4/NKG2A (AYA22T) aptamers demonstrated in silico binding to sequences of the CTLA4 protein, NKG2A protein, and CD94/NKG2A heterodimer protein complex (Figure 1).

### 4.2. Binding Specificity Assessment of CTLA4/NKG2A Aptamers (AYA22T) to CTLA4 and NKG2A Proteins

**Figure 2:**
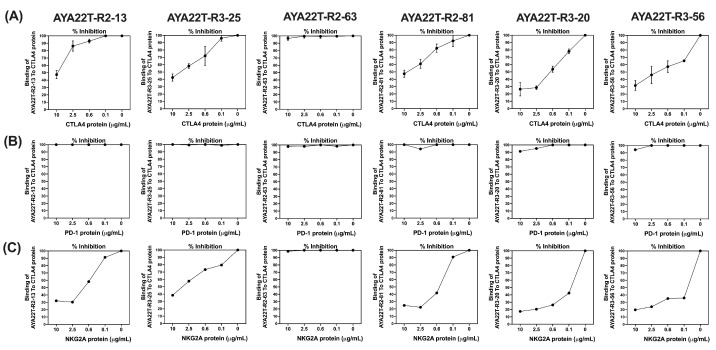
Assessment of the competitive specificity of in silico-developed AYA22T-aptamers to CTLA4/NKG2A in the presence of CTLA4, NKG2A, and PD-1 proteins. An ELISA-based competition assay was conducted to determine the specificity of binding for AYA22T-aptamers (AYA22T-R2-13, AYA22T-R3-25, AYA22T-R2-81, AYA22T-R3-20, AYA22T-R3-56, and AYA22T-R2-63) to CTLA4 protein (5.0 µg/mL) immobilized on a MaxiSorp plate. Biotinylated CTLA4 aptamers at a concentration of 5.0 µM were introduced in the presence of varying concentrations of (**A**) CTLA4, (**B**) PD-1, or (**C**) NKG2A proteins. The binding of the aptamer was detected using streptavidin-HRP and assessed via ELISA. The absorbance of each well was measured at 450 nm using an automatic microplate reader. Percent inhibition was calculated as follows: percent inhibition = [1− (A_inhibitor_/A_uninhibitor_) ] × 100.

We aimed to characterize the binding specificity of computationally designed AYA22T aptamers toward the CTLA4 and NKG2A protein. Figure 2 delineates the competitive specificity assessment of AYA22T aptamers concerning the surface receptor proteins—CTLA4, PD-1, and NKG2A expressed on cytotoxic CD8 T cells (CTLs). Utilizing the competition ELISA method, we observed specific binding of five AYA22T aptamers to CTLA4 and NKG2A, while none exhibited binding specificity towards PD-1. In Figure 2A, we observed approximately 55% binding specificity for AYA22T-R2-13, 60% for AYA22T-R3-25, 55% for AYA22T-R2-81, 75% for AYA22T-R3-20, and 70% for AYA22T-R3-56, respectively, towards CTLA4. Remarkably, in Figure 2C, a high degree of specific binding—approximately 70% for AYA22T-R2-13, 65% for AYA22T-R3-25, 75% for AYA22T-R2-81, 80% for AYA22T-R3-20, and 80% for AYA22T-R3-56, respectively, towards NKG2A was observed. However, compared to CTLA4 and NKG2A, none of the AYA22T aptamers demonstrated binding specificity towards PD-1 protein (Figure 2B). Subsequently, we investigated the binding specificity of the selected five AYA22T aptamers towards CTLA4 and NKG2A proteins. We observed that the presence of equimolar concentrations of CTLA4 or NKG2A in the aptamer mixture inhibited the binding of aptamers to the CTLA4 protein and NKG2A (Supplementary Materials, Figure S1A,B). This competency assessment revealed superior specificity of AYA22T-R2-13 and AYA22T-R3-25 towards the CTLA4 protein. However, all AYA22T aptamers, especially AYA22T-R2-13, AYA22T-R3-25, and AYA22T-R2-81, exhibited stronger binding towards the NKG2A protein. We further elucidated the binding specificity of the AYA22T-R2-13 aptamer to the CTLA4 protein using a concentration-dependent direct binding ELISA, as illustrated in Supplementary Materials, Figure S2. Notably, we observed exclusive binding of AYA22T-R2-13 to the CTLA4 protein compared to BSA and D-dimer proteins.

### 4.3. Detection of AYA22T Aptamers’ Binding to CTLA4 Protein

**Figure 3:**
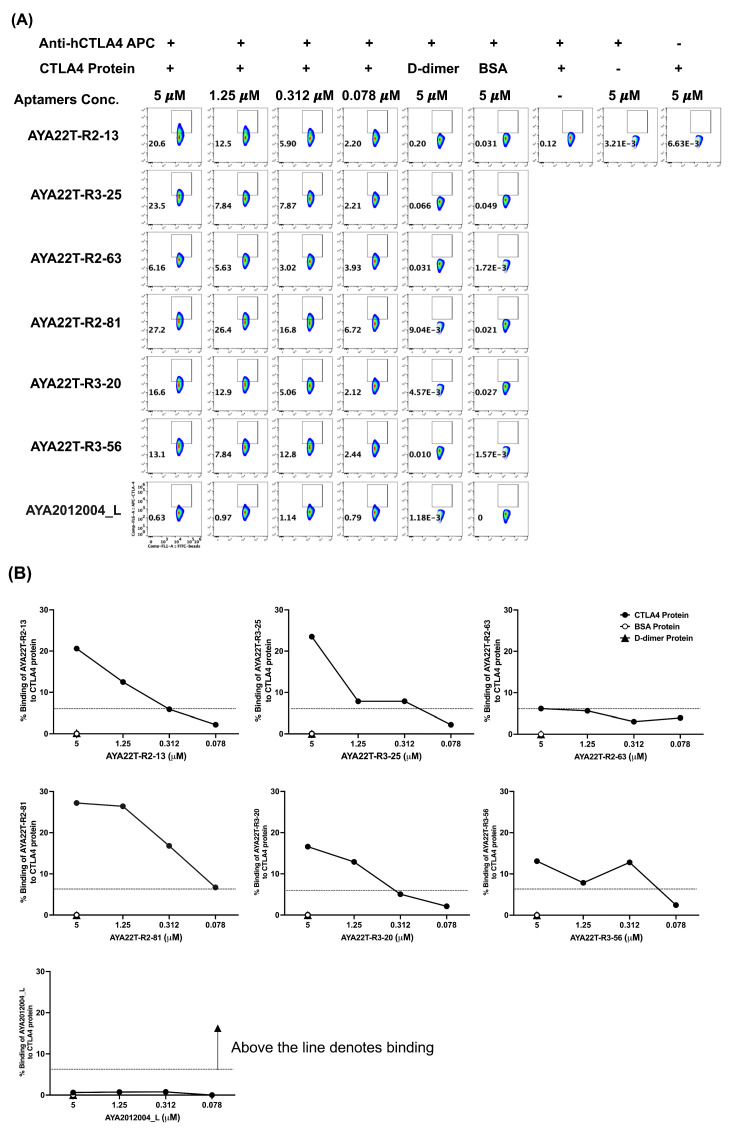
Binding assessment of AYA22T-aptamers to CTLA4 protein by bead-based flow cytometry. The experimental process involved the initial washing of streptavidin-coated fluorescent particles with a manufacturer-recommended wash buffer, followed by the preparation of a particle working concentration in the same buffer. The prepared particles were then incubated with biotinylated aptamers (AYA22T-aptamers) and control aptamer 2 (AYA2012004_L) to allow binding and capture, followed by centrifugation and subsequent washing steps. Subsequently, wells were loaded with recombinant CTLA-4 protein, incubated, and subjected to further washing before being treated with APC-labeled anti-CTLA-4 surface marker. The resulting complex underwent final washing steps and was analyzed through acquisition using the Navios^TM^EX Flow Cytometer (Beckman Coulter Inc., Brea, CA, USA). Flow cytometry data were analyzed using FlowJo^TM^v10.8.1 (Becton Dickinson Life Sciences, Franklin Lakes, NJ, USA). (**A**,**B**) depicts the frequency of percent (%) binding of AYA22T-aptamers. All data are representative of at least three independent experiments.

Subsequently, to validate the specific binding affinity of AYA22T aptamers towards the CTLA4 protein, we conducted an assessment utilizing streptavidin-coated fluorescent particles analyzed through flow cytometry (Figure 3A,B). Biotinylated AYA22T aptamers were employed at various concentrations in conjunction with streptavidin-coated fluorescent particles, and their binding was evaluated in the presence and absence of CTLA4 protein, along with control proteins, D-dimer and BSA. Detection was carried out using an anti-human CTLA4 APC-conjugated antibody. Our observations indicated that, with the exception of AYA22T-R2-63, all other AYA22T aptamers exhibited binding to the CTLA4 protein. Notably, AYA22T-R2-13, AYA22T-R3-25, AYA22T-R2-81, and AYA22T-R3-20 demonstrated superior binding, with more pronounced bindings observed at higher aptamer concentrations. These findings collectively underscore the specific and varying binding affinities of these aptamers towards the CTLA4 protein.

### 4.4. Detection of AYA22T Aptamers’ Binding to CD8 T Cells and NK Cells

**Figure 4:**
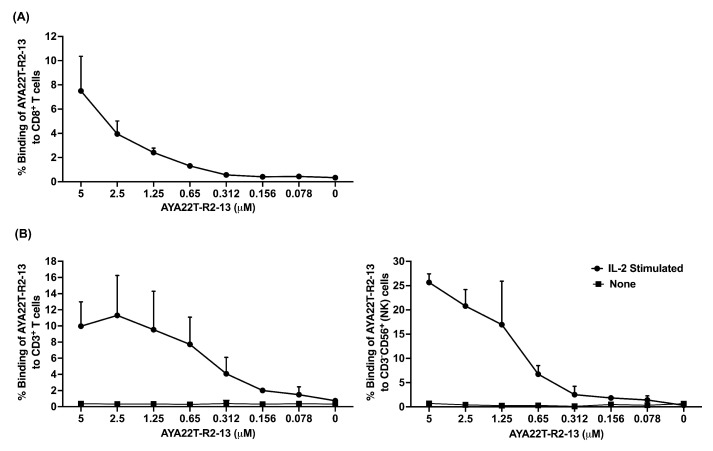
Binding of AYA22T-R2-13 to the cell surface of enriched CD8^+^ T cells, CD3^+^ T cells, and CD3^−^CD56^+^ (NK) cells in PBMCs. (**A**) Assessment of AYA22T-R2-13 binding to IL-2 (20 ng/mL for 48 h)-activated CD8^+^ T cells. (**B**) The presence or absence of IL-2 (20 ng/mL) activation in PBMCs from healthy donors was determined to observe the assessment of AYA22T-R2-13 binding to CD3^+^ T cells and CD56^+^CD3^−^ (NK) cells. Cells were stained with serially diluted biotinylated AYA22T-R2-13 and detected using SA-APC (added at a 1:50 dilution in FACS buffer). Fixable viability dye eFluor 450 was used for cell staining, followed by three washes with FACS buffer. Subsequently, cells were fixed by adding 300 μL/well of FluoroFix buffer before analysis. Gating was performed by initially selecting cells based on forward scatter and side scatter, followed by the selection of singlets and live cells. The Navios EX flow cytometer (Beckman Coulter) was used for the flow cytometry experiments, and FlowJo v10.8.1 (Becton Dickinson Life Sciences) was employed for the analysis of flow cytometry data. All data are representative of at least three independent experiments. Error bars represent mean ± SD.

We explored the binding capability of AYA22T aptamers to native proteins expressed on CD8 T cells and NK cells. Initially, we investigated whether activated CD8 T cells and NK cells express the CTLA4 protein and NKG2A protein, respectively. Our observations confirmed significant expression of CTLA4 in IL-2-activated CD8 T cells, as demonstrated by detection using the anti-human CTLA4 APC-conjugated antibody (Supplementary Materials, Figure S3A) and IL-2+IL-15-activated NK cells, as demonstrated by detection using the anti-human NKG2A PE and CD94 AF700-conjugated antibodies (Supplementary Materials, Figure S3B). This finding is consistent with the observations reported by Wang et al. in 2002 [35]. Conversely, we could not detect any binding of the anti-human CTLA4 APC antibody to IL-2-activated 293T cells (negative control cells), confirming the specificity of the antibody used (Supplementary Materials, Figure S4B). To ascertain the binding specificity of AYA22T aptamers to the CTLA4 protein, we conducted staining of IL-2-activated 293T cells with biotinylated AYA22T aptamers, including control aptamers. Intriguingly, neither the AYA22T aptamers nor the control aptamers exhibited any binding to the 293T cells (Supplementary Materials, Figure S4A). These binding results of our AYA22T aptamers were consistent with those obtained using commercially available anti-human CTLA4 antibodies (Supplementary Materials, Figure S4A,B). Subsequently, we investigated the binding of AYA22T aptamers to IL-2-activated enriched human CD8 T cells (Figure 4A), total CD3+ T cells (Figure 4B and Supplementary Materials, Figure S5A), and CD3-CD56+ (NK) cells (Figure 4C and Supplementary Materials, Figure S5B). Notably, AYA22T-R2-13 exhibited dose-dependent binding to CD8 T cells, total T cells, and NK cells. Importantly, our findings revealed that AYA22T-R2-13 exclusively binds to activated T cells and NK cells (Supplementary Materials, Figure S5A,B), displaying no binding to unstimulated T cells and NK cells (Supplementary Materials, Figure S6A,B). Furthermore, akin to AYA22T-R2-13, AYA22T-R3-25 exhibited binding to activated T cells and NK cells; however, AYA22T-R2-81, AYA22T-R3-20, and AYA22T-R3-56 did not demonstrate binding to T cells and NK cells (Supplementary Materials, Figure S7A,B). These findings underscore the distinctive binding affinity of AYA22T-R2-13 and AYA22T-R3-25 towards activated T cells and NK cells, emphasizing their specificity in binding to these immune cell subsets.

### 4.5. Specificity of Binding of AYA22T-R2-13 to CTLA4 and NKG2A on Activated CD8 T Cells

**Figure 5:**
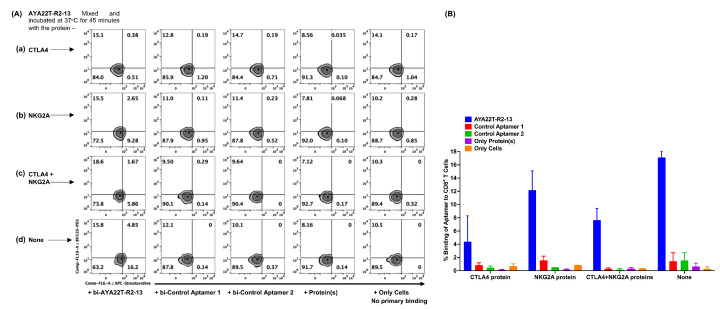
Decrease in the binding of AYA22T-R2-13 to the CTLA4 receptor protein expressed on activated CD8 T cells due to competitive binding in the presence of CTLA4 or NKG2A. CD8 T cells (N = 2) were enriched from two different healthy donors’ PBMCs and cultured in the presence of IL-2 for 24 h. The AYA22T-R2-13 binding to CTLA4 surface protein on CD8 T cells was determined using a competition method. Biotinylated AYA22T-R2-13 was mixed with CTLA4 protein, NKG2A, and a combination of CTLA4 and NKG2A, followed by incubation at 37 °C for 45 min. Subsequently, CD8 T cells were incubated for 30 min at 4 °C. The cells were then washed twice with a solution of 2% FBS and 2 mM EDTA, stained with anti-human PD-1 BV510 and streptavidin-APC, including a fixable viability dye, at 4 °C for 30 min. After washing, the cells were acquired using the Navios EX flow cytometer (Beckman Coulter). FlowJo v10.8.1 (Becton Dickinson Life Sciences) was employed for the analysis of flow cytometry data. (**A**,**B**) depicts the frequency of percent (%) binding of aptamer to CD8^+^ T cells. All data are representative of at least three independent experiments. Error bars represent mean ± SD.

To ascertain the specific binding of AYA22T-R2-13 to cell surface-expressed CTLA4 protein, we conducted a competitive binding assay. Biotinylated AYA22T-R2-13 was incubated at 37 °C for 45 min with or without CTLA4, NKG2A, and in combination with CTLA4 and NKG2A, respectively. Subsequently, the mixture was added to IL-2-activated CD8 T cells and subjected to staining with streptavidin-APC. Flow cytometry analysis was performed as depicted in Figure 5. Our findings revealed that approximately 16% of CD8 T cells exhibited binding with AYA22T-R2-13. However, this binding percentage of CD8 T cells to AYA22T-R2-13 was noticeably inhibited in the presence of competitive proteins, namely CTLA4, NKG2A, or a combination of CTLA4 and NKG2A. These observations suggest that the binding of AYA22T-R2-13 to CTLA4/NKG2A on activated CD8 T cells is specific, as evidenced by the reduction in binding percentage in the presence of competitive CTLA4, NKG2A, or their combination.

### 4.6. Detection of Cell Surface Binding of AYA22T-R2-13 via Competitive Co-Staining Binding Inhibition Assay

**Figure 6:**
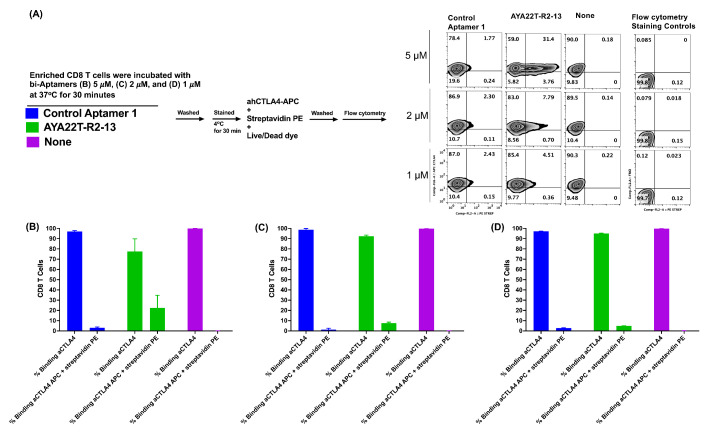
Assessment of AYA22T-R2-13 binding to native cell surface CTLA-4 receptor on CD8 T cells. (**A**) Enriched IL-2-activated CD8 T cells were exposed to biotinylated AYA22T-R2-13 or a control aptamer-1 at (**B**) 5 µM, (**C**) 2 µM, and (**D**) 1 µM for 45 min at 4 °C. Following incubation, cells were stained with streptavidin-APC, APC-labeled anti-human CTLA4, and fixable viability dye. Cells were then incubated for 30 min at 4 °C. Afterward, the stained cell samples were acquired using the Navios EX flow cytometer (Beckman Coulter) and subsequently analyzed with FlowJo v10.8.1 (Becton Dickinson Life Sciences) to investigate the co-staining binding of AYA22T-R2-13 to CTLA4 receptor on CD8 T cells. All data are representative of at least three independent experiments. Error bars represent mean ± SD.

The competitive inhibition binding assay was employed to evaluate the interaction of AYA22T-R2-13 with the native CTLA-4 receptor expressed on IL-2-activated CD8 T cells. Figure 6A illustrated that biotinylated AYA22T-R2-13 and a control aptamer were incubated with enriched CD8 T cells at concentrations of 5 µM, 2 µM, and 1 µM for 45 min and then subsequently stained with SA-APC, APC-labeled anti-human CTLA4, and fixable viability dye. Flow cytometry analysis revealed in Figure 6C,D that the co-staining binding of AYA22T-R2-13 to the CTLA4 receptor on CD8 T cells was observed 31%, 7%, and 4% on 5 µM, 2 µM, and 1 µM AYA22T-R2-13 incubation, respectively. Interestingly, 5 µM AYA22T-R2-13 primary incubation with CD8 T cells competitively inhibited the binding of anti-human CTLA4 monoclonal antibody (Figure 6B). The results from this competitive inhibition binding assay provide crucial insights into the specificity and potency of AYA22T-R2-13 in targeting the CTLA-4 receptor on activated CD8 T cells, laying the foundation for further investigations into the therapeutic potential of this dual CTLA4/NKG2A aptamer.

### 4.7. Assessment of Cytotoxicity of AYA22T-R2-13 on Tumors and Immune Cells

**Figure 7:**
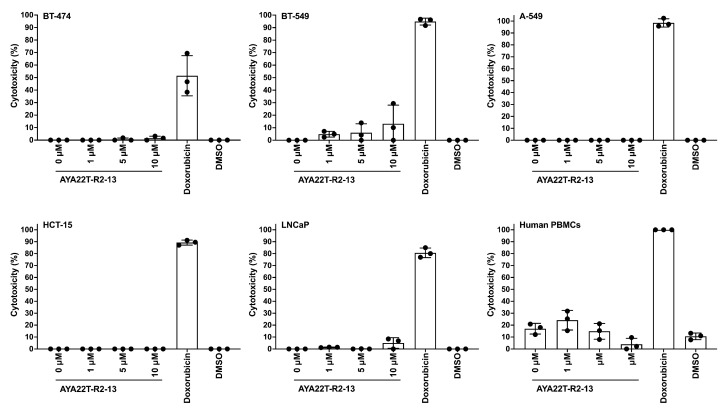
Aptamer cytotoxicity assessment using Sulforhodamine B (SRB) assay. A quantity of 2 × 10^4^ tumor cells or PBMCs per well in 200 µL of cell-culture medium containing 10% FBS. The cells were allowed to adhere for 24 h at 37 °C with 5% CO_2_ before conducting the cell viability experiments in 96-well flat-bottom microtiter plates. Aptamers were applied at concentrations of 0 µM, 1 µM, 5 µM, and 10 µM, alongside a positive control (doxorubicin, 1 µL of 20 mM) and DMSO. These compounds were incubated with the cells in the medium for 72 h at 37 °C with 5% CO_2_. Following incubation, the cells were fixed, stained using the SRB cell cytotoxicity assay kit, and then subjected to the solubilization method recommended by the kit. Absorbance was measured at 565 nm using a plate reader. Each experiment was performed in triplicate for accuracy and reproducibility. Error bars represent mean ± SD.

We conducted an investigation to determine the cytotoxic potential of AYA22T-R2-13 and its impact on cell viability. Utilizing the Sulforhodamine-B (SRB) assay, we evaluated the cytotoxic effect of various concentrations of AYA22T-R2-13 on different tumor cells and human peripheral blood mononuclear cells (PBMCs), as depicted in Figure 7. Cells were subjected to incubation with and without AYA22T-R2-13, along with doxorubicin and DMSO, employed as positive and negative controls, respectively. Our results indicate that treatment with AYA22T-R2-13 did not induce a reduction in the viability of tumor cells across various doses. Notably, the cell viability of aptamer-treated cells remained comparable to both untreated cells as well as those treated with DMSO. In contrast, the positive control, doxorubicin, demonstrated increased cytotoxicity towards both tumor cells and human PBMCs. These findings suggest that AYA22T-R2-13 does not exhibit cytotoxic effects on tumor cells or human PBMCs under the conditions tested, implying its lack of impact on cell viability.

### 4.8. Blockade of the CTLA4-CD80/86 Axis Using AYA22T-R2-13 Unleashes CTL-Mediated Lysis of Tumor Cells In Vitro

**Figure 8:**
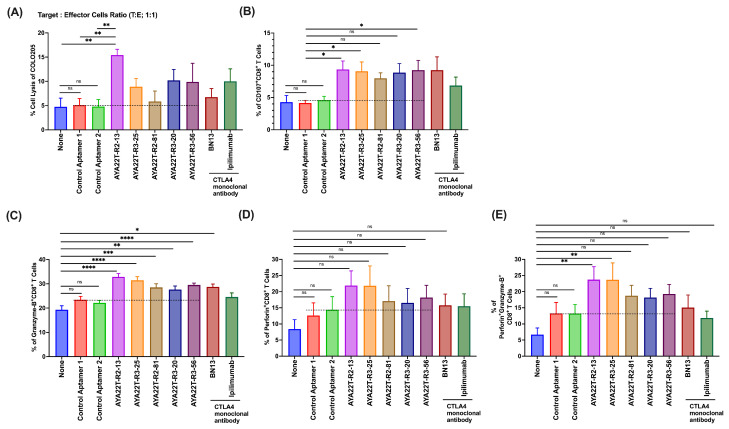
AYA22T-aptamers enhance CD8 T cell cytolytic activity in non-hypermutated colon adenocarcinoma cell line in vitro. Enriched CD8 T cells isolated from healthy donors PBMCs were incubated with recombinant hIL-2 (20 ng/mL) for 24 h. CD8 T cells were then co-cultured with hIFN-γ-stimulated COLO205 tumor cells at a ratio of 1:1 (T:E; target:effector) in the presence or absence of AYA22T-aptamers, control aptamers (negative control), and CTLA4 monoclonal antibodies (positive control) for 24 h. (**A**) Percent cell killing was assessed by lactate dehydrogenase (LDH) assay. Each dot represents the % cell lysis of COLO205 from individual donors’ CD8 T cells. (**B**) The frequencies of CD107-expressing CD8^+^ T cells. (**C**–**E**) The frequencies of granzyme-B, perforin, and granzyme-B^+^perforin^+^ expressing CD8^+^ T cells. The error bars depict the mean ± SD of CD8 T cells from a total of N = 10 healthy donors, each contributing to an independent experimental set. * *p* < 0.05, ** *p* < 0.01. *** *p* < 0.001. **** *p* < 0.0001.

**Figure 9:**
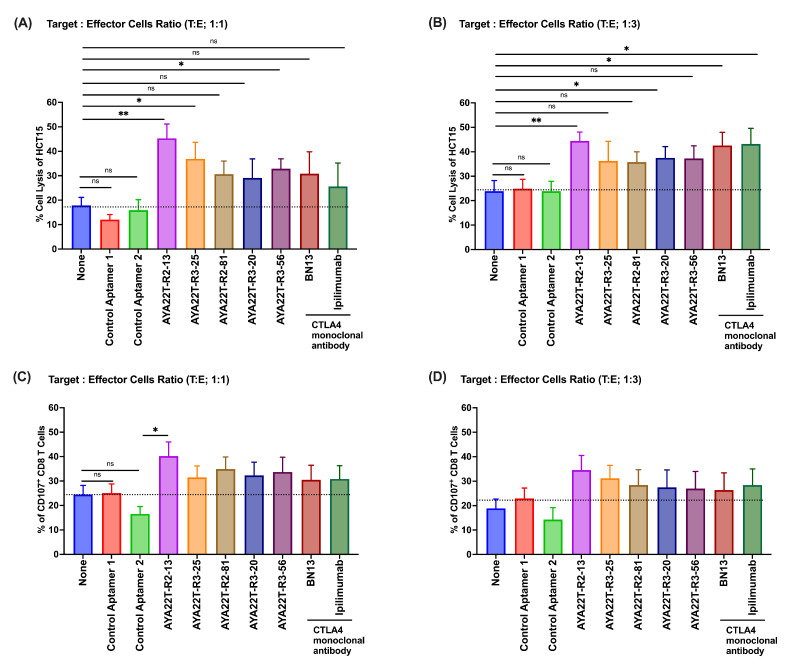
Assessment of AYA22T-aptamers cell cytolytic activity of CD8 T cells in hypermutated colon adenocarcinoma cell line in vitro. AYA22T-aptamers enhance CD8 T cell cytolytic activity in vitro. Enriched CD8 T cells isolated from healthy donors PBMCs were incubated with recombinant hIL-2 (20 ng/mL) for 24 h. CD8 T cells were then co-cultured with hIFN-γ-stimulated HCT15 tumor cells at a ratio of 1:1 or 1:3 (T:E), in the presence or absence of CTLA4 aptamers, control aptamers (negative control), and CTLA4 monoclonal antibodies (positive control) for 48 h. (**A**,**B**) Percent cell killing was assessed by lactate dehydrogenase (LDH) assay. The error bars depict the mean ± SD of CD8 T cells from a total of N = 10 healthy donors, each contributing to an independent experimental set. (**C**,**D**) The frequencies of CD107a-expressing CD8^+^ T cells. The error bars depict the mean ± SD of CD8 T cells from a total of N = 4 healthy donors, each contributing to an independent experimental set. * *p* < 0.05, ** *p* < 0.01. Ns in the figure indicates non-significant.

**Figure 10:**
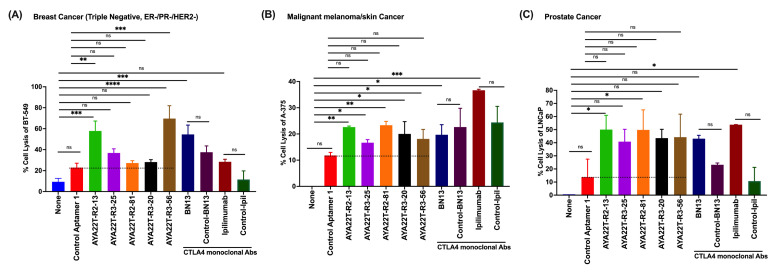
Assessment of AYA22T-aptamers cell cytolytic activity of CD8 T cells in various tumor cell lines in vitro. AYA22T-aptamers (CTLA4/NKG2A aptamers) enhance CD8 T cell cytolytic activity in vitro. Enriched CD8 T cells isolated from healthy donors PBMCs were incubated with recombinant hIL-2 (20 ng/mL) for 24 h. CD8 T cells were then co-cultured with hIFN-γ-stimulated tumor cells ((**A**) BT-549, (**B**) A-375, and (**C**) LNCaP), at a ratio of 1:1 (T:E), in the presence or absence of CTLA4/NKG2A aptamers, control aptamers (negative control), and CTLA4 monoclonal antibodies (positive control) for 48 h. Percent cell killing was assessed by lactate dehydrogenase (LDH) assay. The error bars depict the mean ± SD of CD8 T cells from a total of N = 2 healthy donors, each contributing to an independent experimental set. * *p* < 0.05, ** *p* < 0.01. *** *p* < 0.001. **** *p* < 0.0001. Ns in the figure denotes non-significant.

Upon establishing the binding specificity of AYA22T-R2-13 to CTLA-4 on cytotoxic T lymphocytes (CTLs), our investigation aimed to uncover its potential in enhancing CTL-mediated tumor cell lysis by disrupting CTLA-4 interactions with the CD80 or CD86 axis. Initially, we probed the expression levels of CD86 and CD80 essential molecules for CTLA-4 interaction on tumor cells. Following stimulation of HCT-15 cells with IFN-γ, we observed heightened expression of CD86 and PD-L1 (Supplementary Materials, Figure S8A,C), aligning with previously reported findings [36,37,38]. However, we did not observe CD80 expression on tumor cells. Further experiments involved the enrichment of human CD8 T cells cultured in the presence of recombinant hIL-2 for 24 h, followed by assessment of their cytolytic activity against various tumor cell lines, COLO205, HCT-15, BT-549, BT-474, A-375, and LNCaP, as targets. Evaluation of AYA22T-aptamer at different concentrations revealed their efficacy in inducing CTL-mediated lysis of specific tumor cells, particularly BT-549 (ER-/PR-/HER2-) (Supplementary Materials, Figure S9A), when compared to control aptamers or no treatment. Subsequently, a 2 µM dose of AYA22T aptamers was established for subsequent experiments. Figure 8A vividly depicted the heightened killing efficacy of CD8 T cells in the presence of AYA22T-R2-13, manifesting significantly greater lysis of non-hypermutated colorectal tumor cells (COLO205) in contrast to control aptamers and the mock condition. To assess AYA22T-R2-13’s inhibitory effect on effector cells, we monitored CD107a surface expression on CD8 T cells co-cultured with COLO205 tumor cells. The introduction of AYA22T-R2-13 notably amplified CD107a production by CD8 T cells, surpassing levels observed with control aptamers and the mock condition (Figure 8). Moreover, treatment with AYA22T-R2-13 led to increased frequencies of granzyme-B^+^ (Figure 8C), perforin^+^ (Figure 8D), and double-positive granzyme-B^+^perforin^+^ (Figure 8E) CD8^+^ T cells compared to control aptamers and the mock condition. Subsequently, we investigated whether AYA22T-R2-13 could induce CTL-mediated lysis of hypermutated colorectal tumor cells using the HCT-15 cell line. Figure 9A,B demonstrated significant induction of CD8 T cell-mediated HCT-15 cell lysis at varying target–effector ratios upon application of AYA22T-R2-13. This was further bolstered by a marked increase in the production of CD107a^+^CD8 T cells compared to control aptamers and the mock condition (Figure 9C,D). Additionally, other AYA22T aptamers showcased heightened CTL-mediated tumor cell lysis, although their effects on CTLs were not significantly different from monoclonal antibodies BN13 and ipilimumab (Figure 8 and Figure 9). In our subsequent investigation, we aimed to evaluate whether AYA22T-R2-13 could facilitate CTL-mediated lysis of breast cancer, malignant melanoma/skin cancer, and prostate cancers. Figure 10 demonstrates our use of BT-549 (Figure 10A), A-375 (Figure 10B), and LNCaP (Figure 10C) tumor cell lines for this purpose. Notably, AYA22T-R2-13 significantly induced CD8 T cell-mediated lysis of tumor cells compared to the control aptamer and mock condition. Additionally, our other AYA22T aptamers exhibited induced CTL-mediated tumor cell lysis, akin to the effects observed with positive controls, monoclonal antibodies, BN13, and ipilimumab. Further, we investigated the stability and functionality of aptamers in the presence or absence of human serum. As illustrated in Supplementary Materials, Figure S10, aptamers AYA22T-R2-13, AYA22T-R3-25, AYA22T-R2-81, control aptamer, and the untreated (no treatment) conditions were first incubated either with or without undiluted human serum. Subsequently, these samples underwent effector:target cell co-culture, 1:1. Our observations revealed that the cytotoxicity (%) of HCT-15 cells by CD8 T cells, whether in the presence or absence of human serum, did not compromise the functional properties of AYA22T-R2-13, AYA22T-R3-25, and AYA22T-R2-81. It remained highly effective in promoting CTL (cytotoxic T lymphocyte) activity. In assessing AYA22T-aptamer stability under physiological conditions, we conducted in vitro serum incubation (1, 3, 6, and 16 h at 37 °C). Gel electrophoresis revealed nearly intact AYA22T-aptamers up to 6 h, with minimal degradation (Supplementary Materials, Figure S11). However, a shift occurred between 6 and 16 h, showing four of five aptamers with faint or degraded bands, indicating structural changes. Despite degradation, the serum half-life within the initial 6 h remained acceptable. This addresses concerns of short aptamer half-life, indicating manageability. The serum stability results support pursuing in vivo studies with AYA22T-aptamers. Even pre-modification, AYA22T-aptamers demonstrate notable stability, emphasizing therapeutic potential in animal studies. These findings encourage further investigations and optimization for harnessing AYA22T-aptamers’ therapeutic benefits. In summary, our study validates the binding of AYA22T-R2-13 to CTLA-4, thereby amplifying CTL-mediated tumor lysis.

### 4.9. AYA22T-R2-13 Unleashes NK Cell-Mediated Lysis of Tumor Cells In Vitro

**Figure 11:**
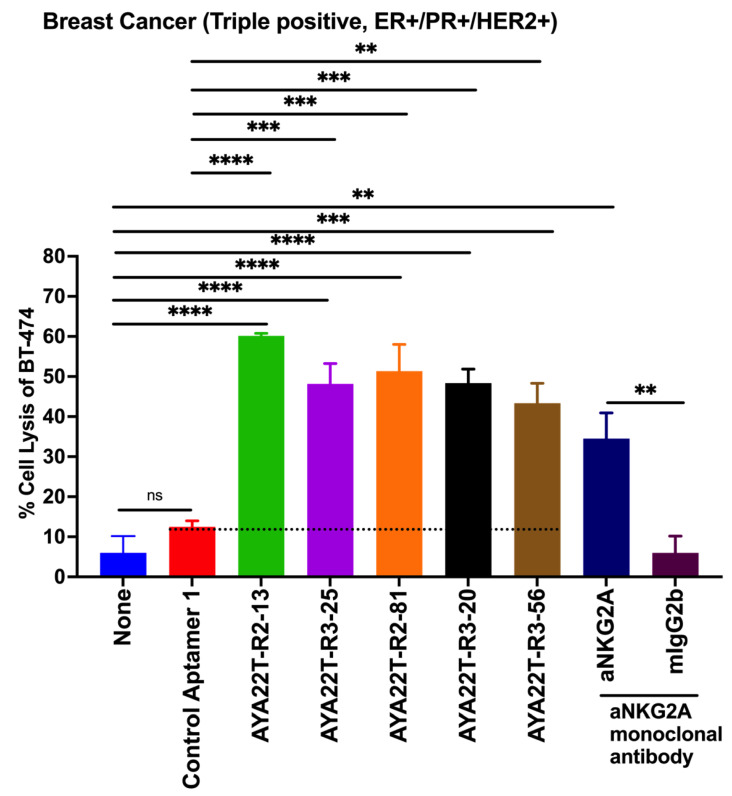
AYA22T-aptamers (CTLA4/NKG2A aptamers) enhance NK cell cytolytic activity in vitro. Enriched NK cells isolated from healthy donors’ PBMCs were incubated with recombinant hIL-2 (20 ng/mL) for 24 h. NK cells were then co-cultured with hIFN-γ-stimulated tumor cells, BT-474 at a ratio of 1:3 (T:E) in the presence or absence of CTLA4/NKG2A aptamers, control aptamer (negative control), and anti-human NKG2A monoclonal antibodies (positive control) for 48 h. Percent cell killing was assessed by lactate dehydrogenase (LDH) assay. The error bars depict the mean ± SD of CD8 T cells from a total of N = 3 healthy donors, each contributing to an independent experimental set. ** *p* < 0.01. *** *p* < 0.001.**** *p* < 0.0001. Ns in the figure devotes non-significant.

We assessed AYA22T-aptamers’ binding specificity to NKG2A on NK cells, aiming to enhance NK cell-mediated tumor lysis by disrupting NKG2A interactions with the HLA-E axis. Expression levels of HLA-E and PD-L1 on BT-549, BT-474, LNCap, A-375, A-549, and HCT-15 cells were probed. Elevated HLA-E and PD-L1 expression was observed (Supplementary Materials, Figure S8A–C), consistent with reported findings [11,25,26,30]. Further, our focus shifted to evaluating the potential of AYA22T-aptamers in inducing NK cell-mediated lysis of tumor cells. We evaluated the efficacy of AYA22T-aptamer on NK cell medicated tumor cell lysis at different concentrations (Supplementary Material, Figure S9B) and select 2 µm dose for subsequent experiments. Using BT-474, HCT-15, and BT-549 human tumor cell lines (depicted in Figure 11 and Supplementary Materials, Figure S12A,B), we observed significant NK cell-mediated lysis of tumor cells, distinct from the control aptamer and mock condition. Furthermore, our other AYA22T aptamers induced similar NK cell-mediated tumor cell lysis, resembling the effects seen with positive controls, such as the monoclonal antibody anti-NKG2A. This study underscores the confirmed binding of AYA22T-aptamers to NKG2A on NK cells, amplifying NK cell-mediated tumor lysis.

## 5. Discussion

The rationale behind our aptamer design is rooted in the documented effectiveness of targeting NKG2A and CTLA4 for bolstering anti-tumor immunity [39,40,41]. This strategic combination leverages the well-established impact of immune checkpoint blockade (ICB) on immune regulation. Our approach is consistent with the evolving paradigm of combination ICB therapies, which have exhibited promising clinical outcomes by addressing the intricate network of immune checkpoints. By concurrently targeting NKG2A [13,42] and the well-established modulator CTLA-4 [43,44] in T cell responses, our aptamer seeks to augment the anti-tumor immune response, presenting a comprehensive approach to dual immune checkpoint blockade.

In this study, a computational biology approach was employed to engineer a series of enriched aptamers targeting CTLA-4 and NKG2A receptors on CD8^+^ T cells and NKG2A receptors on NK cells interactions with B7-1/B7-2 and HLA-E on tumor cells. Among these aptamers, AYA22T-R2-13 exhibited superior performance, demonstrating a dose-dependent inhibition of CTLA-4/NKG2A function. Importantly, our in vitro experiments unveiled AYA22T-R2-13’s dual mechanism of action. This aptamer activated T cells by impeding the CTLA-4 interaction with B7-1/B7-2, enhancing the eradication of cancer cells, and augmenting NK cells’ capacity for detecting and eliminating HLA-E-expressing cancer cells by inhibiting NKG2A. Moreover, AYA22T-R2-13 displayed potent CD8^+^ T/NK cell-mediated tumor cell lysis [33,42] across various cancer cell lines, including colorectal, breast, melanoma/skin, and prostate cancer. Our findings highlight the potential of AYA22T-R2-13 as a viable alternative to antibody-based immunotherapy, offering advantages such as heightened specificity, reduced toxicity and immunogenicity, and cost-effective production.

Numerous studies have elucidated the intricacies of anti-CTLA-4 therapy, with a particular emphasis on Treg cell involvement and unanticipated outcomes in CD8^+^ effector cells. These investigations collectively establish that the primary targets of anti-CTLA-4 therapy encompass Treg cells and antigen-presenting cells (APCs). Mechanistically, the blockade of CTLA-4 disrupts Treg cell immunosuppressive functions. Anti-CTLA-4 monoclonal antibodies (mAb) further induce potent anti-tumor effects by depleting CTLA-4-expressing Treg cells within the tumor microenvironment (TME) through Ab-dependent cytotoxic activity (ADCC). The pivotal role of Treg cells in tumor resistance and the subsequent enhancement of anti-tumor immunity upon their suppression underscore the therapeutic significance of anti-CTLA-4. While the well-established role of CTLA-4 in Tregs has been documented [45,46], recent insights from Hegel et al. [47] delineate a specific association between CTLA-4 and the transcription factor Eomes in CD8^+^ T-cell function. In contrast to conventional understanding, Hegel et al. present evidence that CTLA-4 selectively targets Eomes in CD8^+^ effector T cells, challenging the prevailing notion of a generalized inhibition by CTLA-4 on downstream regulators. This connection is underscored by observed reductions in IFN-γ and granzyme-B expression, coupled with an augmentation of cytolytic function in CTLA4^−/−^CD8^+^ T cells. Incorporating insights from Hegel et al. [47], our manuscript contributes to an enriched understanding of CTLA-4’s role. We extend the investigation beyond cell cytotoxicity via LDH assay, delving into granzyme-B [48] and perforin, alongside CD107a expression [49]. These findings underscore that the heightened anti-tumor effects observed in anti-CTLA-4 therapy align with Hegel et al.’s specific modulation of CD8^+^ effector cells through Eomes targeting. Consequently, our study seeks to evaluate the impact of anti-CTLA4/NKG2A aptamers on immune checkpoint interactions, specifically examining CTLA4-CD80/CD86 and NKG2A-HLA-E interactions between CD8 T cells/NK cells and tumor cells.

Current research on immune checkpoints, such as CTLA-4, PD-1, and PD-L1, has shown remarkable clinical benefits. Our initial goal was to design an anti-CTLA-4/NKG2A aptamer with low immunogenicity, low toxicity, and stable binding [50], specifically targeting CTLA-4/NKG2A on both T cells and NK cells. The investigation into the effects of CTLA-4 on T cells binding tumor cells holds significant importance due to the pivotal role of CTLA-4 in regulating immune responses. CTLA-4, as a homolog of CD28, competes for binding with surface antigens B7-1 (CD80) and B7-2 (CD86) upon T cell activation. This competition attenuates CD28 co-stimulation and inhibits signaling, influencing the host immune response. The interaction between CTLA-4 and B7 is a critical checkpoint that, when blocked, reshapes the immune response and demonstrates sustained anti-tumor effects in specific cancer subpopulations [51,52,53,54,55]. The blockade of CTLA-4, along with the PD-1/PD-L1 axes, constitutes the foundation of current cancer immunotherapy [56].

We are excited to report the specific binding of our aptamers to CTLA-4 and NKG2A, marking a significant discovery. Its dual functionality enhanced both NK and CD8 T cell cytotoxic activity against various tumor cell lines while blocking the CTLA-4-B7-1/B7-2 and NKG2A-HLA-E interactions. Simultaneously, we evaluated aptamer binding to PD-1, yet neither AYA22T-R2-13 nor other aptamer candidates showed binding affinity to PD-1 protein. AYA22T-R2-13 distinguished itself from the dual-functional aptamer in Du’s study [21], which combined a CTLA-4 aptamer with a PD-1 aptamer. An inherent challenge in treating tumors is somatic mutations, which represent a key target of anti-tumor immunity [57].

Recently, Huang et al. [20] demonstrated in in vivo studies that aptCTLA-4 treatment significantly increases the number of tumor-infiltrating lymphocytes (CD45^+^) and the percentage of cytotoxic T lymphocytes (CTLs) (CD45^+^ and CD8^+^), as observed in flow cytometry analyses. Immunohistochemistry studies further validate the rise in CD8^+^ cells in tumors treated with aptCTLA-4. These preclinical findings highlight the translational potential of CTLA-4 inhibiting aptamers in modulating the immune response within the tumor microenvironment. Our in vitro findings with the anti-CTLA4/NKG2A aptamer align with the in vivo observations made by Huang et al. [20] and underscore the efficacy of our aptamer in facilitating CTLs targeted tumor lysis.

Immune responses may fail to discern favorable from unfavorable mutations, leading to the production of non-functional antibodies [58]. Our study delved into the prevalence of tumor mutation in breast cancer according to Wagle’s team research [1]. High tumor mutation is present in 5% of all breast cancer cases, and hypermutated breast cancers are more likely to respond to PD-1 inhibitors [59]. Our evaluation of AYA22T-R2-13’s cell cytolytic activity in hypermutated (HCT-15) and non-hypermutated tumor cell lines (COLO205) [60] demonstrated its significant enhancement of CD8^+^ T cell and NK cell cytolytic activity in vitro. Moreover, compared to Ipilimumab and BN13 (anti-human CTLA4 mAb), as well as anti-human NKG2A mAb, AYA22T-R2-13 exhibited improved CD8^+^ T cell and NK cell cytolytic activity, while showing no cytotoxicity in BT-474, BT-549, A-549, HCT-15, LNCaP, and immune cells.

Previously studied CTLA-4 aptamers were generated using the SELEX (Systematic Evolution of Ligands by Exponential Enrichment) method or by combining two aptamers [21]. In contrast, AYA22T-R2-13 was developed utilizing a comprehensive computational approach that predicted secondary and tertiary structures and employed docking simulations to explore interactions between the aptamers and CTLA-4 proteins. This approach minimizes chemical use and reagents while maintaining manageable computing costs. Moreover, in silico approaches enable the investigation or modification of aptamer–ligand interaction details more comprehensively than feasible experimentally [61].

In summary, our research underscores the characteristics and anti-tumor activity of AYA22T-R2-13 aptamer. Our findings highlight that this CTLA4/NKG2A dual-functional aptamer enhances the immune response of human CTLs (CD8^+^ T cells) and NK cells against human tumor cell lines. Our next steps will involve a thorough assessment of AYA22T-R2-13’s biofunction and therapeutic efficacy in vivo, evaluating the safety and efficacy of aptamers in a more realistic physiological environment, and monitoring tumor regression and survival rates in animal models. Cancer remains a formidable health challenge, with persistent annual increases witnessed in new cases and mortality rates. Notably, breast, prostate, and uterine cancers are surging. Economically, cancer care bears an overwhelming burden, with costs varying based on cancer phase and treatment specifics. Immunotherapy, including T cell immune-checkpoint inhibitors and CAR-directed T lymphocytes [6], is pivotal in modern oncology, targeting CTLA-4 and PD-1/PD-L1 pathways. Recent research underscores CTLA-4’s diverse role in immune modulation, impacting T cell activation, IDO induction, and T cell proliferation inhibition. Additionally, recognition of the NKG2A axis, interacting with HLA-E on tumor cells, highlights its significance in immune evasion and cancer progression. Current FDA-approved anti-CTLA-4 therapies exhibit promise but face challenges regarding response rates and immune-related adverse events.

## 6. Conclusions

**Figure 12:**
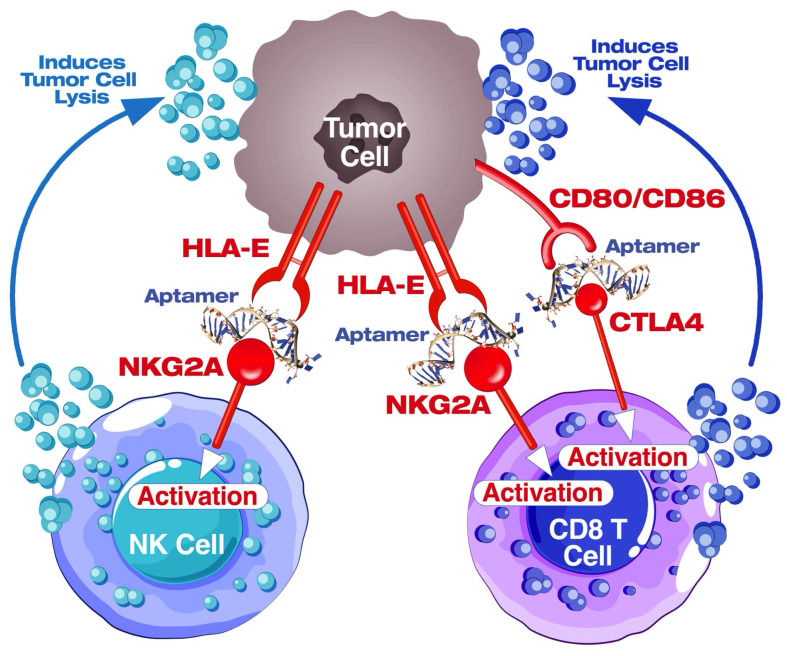
A comprehensive mechanistic representation of the dual CTLA4/NKG2A aptamer (AYA22T) targeting immunotherapy. The diagram highlights the intricate interactions and signaling pathways involved in the aptamer-mediated modulation of immune responses, specifically focusing on the dual targeting of CTLA4 and NKG2A receptors.

In conclusion, the dual-targeting immunotherapy utilizing aptamers against CTLA-4 and NKG2A presents a promising approach for cancer treatment. The strategy aims to unleash the full potential of CD8 T cells and natural killer (NK) cells by concurrently inhibiting immune checkpoint molecules. By targeting CTLA-4 and NKG2A, the aptamers enhance the activation and effector functions of CD8 T cells and NK cells, respectively. This synergistic modulation of two distinct immune pathways may lead to a more comprehensive and potent anti-cancer immune response (Figure 12). The dual aptamer-based approach holds potential for overcoming immune evasion mechanisms employed by cancer cells and represents a novel avenue for developing effective immunotherapeutic strategies against cancer. Further preclinical (in vivo syngeneic tumor model) and clinical studies are warranted to validate the safety, efficacy, and translational potential of this innovative dual-targeting immunotherapy in diverse cancer settings.

## Data Availability

This article has accompanying Supplementary Files. All data generated or analysed during this study are included in this published article (and its Supplementary Materials Files).

## Supplementary Materials

The following supporting information can be downloaded at: https://www.mdpi.com/article/10.3390/cancers16051041/s1, Figure S1. Aptamer Binding to Recombinant CTLA4 Protein; Figure S2. Evaluation of AYA22T-R2-13’s Specific Binding to CTLA4 Protein; Figure S3. CTLA4 expression on activated CD8+ T cells; Figure S4. Detection of Non-Specific Binding of AYA22T-Aptamers on the Cell Surface; Figure S5. Binding of AYA22T-R2-13 to the cell surface of T cells and NK cells; Figure S6. Binding of AYA22T-R2-13 to the cell surface of T cells and NK cells in human PBMCs; Figure S7. Binding of AYA22T aptamers to the cell surface of T cells and NK cells in human PBMCs; Figure S8. Expression of CD86, HLA-E, and PD-L1 by various human tumor cell lines with or without IFN-γ stimulation; Figure S9. Titration of AYA22T-R2-13 dosing concentration for in vitro tumor cell lysis by CD8 T cells and NK cells; Figure S10. Tumor cell lysis activity of AYA22T aptamers by CD8 T cells remains uncompromised in the presence of human serum; Figure S11. CTLA4/NKG2A aptamers (AYA22T) exhibit relative stability in human serum; Figure S12. AYA22T-R2-13 enhances NK cell cytolytic activity in vitro; Table S1: Sequences of CTLA4 aptamers; Table S2: Key resources of materials and methods.

## Abbreviations

The following abbreviations are used in this manuscript:

CTLA-4	cytotoxic T-lymphocyte–associated antigen 4
PD-1	programmed death 1
PD-L1	programmed cell death-ligand 1
IDO	indoleamine 2,3-dioxygenase
NK	natural killer
irAEs	immune-related adverse events
A	adenine
U	uracil
G	guanine
C	cytosine
dU	deoxyuridine
PBMCs	peripheral blood mononuclear cells
LDH	lactate dehydrogenase
