# Supplementary material for: Dual Checkpoint Aptamer Immunotherapy: Unveiling Tailored Cancer Treatment Targeting CTLA-4 and NKG2A
Source: Cancers (Basel). 2024 Mar 4;16(5):1041. doi: 10.3390/cancers16051041 (PMC10931446; doi:10.3390/cancers16051041)
Supplement: Supplementary file 1 [file cancers-16-01041-s001.zip › Supplementary Experimental Method Details.02.09.2024docx.pdf]

Dual Checkpoint Aptamer Immunotherapy: Unveiling Tailored Cancer Treatment Targeting CTLA-4 and NKG2A

EXPERIMENTAL METHOD DETAILS

2.9. Molar to Molar Competition Assay by ELISA

The method for analyzing the binding of aptamers to Human CTLA-4/CD152 and Human NKG2A/CD159a proteins through ELISA was comprised of precise steps. Initially, a MaxiSorp plate was coated overnight with 0.26 µM of Human CTLA-4/CD152 or Human NKG2A/CD159a proteins (Acro Biosystems, Newark, DE, USA) prepared in a 50 mM Carbonate-Bicarbonate solution. Subsequently, the plate was blocked for 1 hour with 2% BSA in 1mM MgCl2 supplemented 1x PBS/Tween. On molar-to-molar concentration, 2 µM aptamer was mixed along with 2 µM CTLA4 protein or NKG2A protein. The mixture was then introduced into the wells. Following this, the plate was incubated at room temperature for 1 hour. For colorimetric detection, HRP-conjugated streptavidin (Thermo Scientific, USA) was utilized in combination with TMB. The reaction was halted by the addition of 1M H_2SO_4, and subsequent color changes were assessed using a spectrophotometer at 450 nm. During the ELISA process, the plate underwent meticulous washing six times with a 200 wash buffer (1mM MgCl2 supplemented 1x PBS/Tween) using a plate shaker set at 900 rpm for 5 minutes after each procedural step, ensuring comprehensive removal of unbound substances and maintaining assay accuracy.

2.11. Flow cytometry-based binding Assay of AYA22T-aptamers to CTLA4 Protein

The investigation of biotinylated aptamer binding to Human CTLA-4/CD152 protein (Acro Biosystems, Newark, DE, USA) via streptavidin-coated fluorescent particles kit was conducted using a meticulous flow cytometry-based approach. Initially, streptavidin-coated fluorescent particles (Spherotech SVFB-2558-6K, Lake Forest, IL, USA) underwent thorough washing with a manufacturer-recommended wash buffer to eliminate 0.02% NaN3. To prepare a working concentration of particles in the wash buffer (comprising 5 mM Tris-HCl (pH 7.5), 0.5 mM EDTA, 1 M NaCl), 2 µL of streptavidin beads per well were utilized. Subsequently, 100 µL of the prepared beads in the wash buffer was dispensed into a 96-well V-bottom plate. Following this, 100 µL of biotinylated aptamers were introduced into the plate and allowed to incubate at room temperature for 1 hour to facilitate the capture of aptamers by the beads. Upon completion of incubation, the plate was centrifuged at 500g for 5 minutes and underwent a wash cycle with the wash buffer. Next, 100 µL of CTLA-4/CD152 protein (Acro Biosystems, Newark, DE, USA), prepared at a concentration of 5 µg/mL in the wash buffer, was dispensed into the wells and incubated for an additional hour at room temperature. The plate underwent subsequent washing and was then incubated for 30 minutes with 50 µL of APC-labeled anti-CTLA-4 surface marker (at a concentration of 1 µL per well). Finally, the plate was washed again and subjected to acquisition in 1x PBS (without CaCl2 & MgCl2) utilizing a flow cytometer for subsequent analysis.

2.12. PBMCs Isolation

The peripheral blood mononuclear cells (PBMCs) were isolated from healthy donors' buffy coats obtained from Carter BloodCare using a Ficoll-Paque Plus density-gradient centrifugation method (Cytiva Life Sciences). In brief, whole blood was diluted at a ratio of 1:3 with 1× PBS containing 1mM EDTA (Sigma-Aldrich, the Netherlands). This diluted blood was carefully layered onto Ficoll-

Dual Checkpoint Aptamer Immunotherapy: Unveiling Tailored Cancer Treatment Targeting CTLA-4 and NKG2A

EXPERIMENTAL METHOD DETAILS

Paque^{PLUS} (GE Healthcare Life Sciences, Sweden) and subjected to density gradient centrifugation (2,000 rpm, 30 mins, acceleration 9, deceleration 0). Following centrifugation, the PBMC layer was collected and washed twice using 1× PBS containing 1mM EDTA, with centrifugation steps at 1,500 rpm for 10 minutes between each wash. Subsequently, the PBMC pellets were resuspended in a freezing medium composed of 90% FBS (HyClone, South Logan, UT, USA) and 10% DMSO (Sigma-Aldrich, the Netherlands). The resuspended cells were then aliquoted into cryo vials, initially placed at -80◦C overnight for gradual cooling, and subsequently transferred to liquid nitrogen for long-term storage until further experimentation.

2.13. Isolation and Stimulation of CD8$^+$ T Cells and NK Cells

The CD8$^+$ T cells and NK cells were isolated and stimulated following specific protocols. Initially, peripheral blood mononuclear cells (PBMCs) were resuspended and washed twice in 2% FBS in 1x PBS containing 1mM EDTA buffer. Subsequently, the isolation of CD8$^+$ T-cells and/or enrichment of NK cells from the PBMC fraction was carried out utilizing the EasySepTM Human CD8$^+$ T cell isolation kit or EasySepTM Human NK Cell Enrichment Kit (Stemcell Technologies, Vancouver, Canada) according to the manufacturer's instructions. Following isolation/enrichment, the CD8$^+$ T cells were subjected to stimulation using recombinant human IL-2 (R&D Systems, Minneapolis, MN, USA) at a concentration of 20 ng/mL and the NK cells were subjected to stimulation using recombinant human IL-2 and human IL-15 (R&D Systems, Minneapolis, MN, USA) at a concentration of 20 ng/mL each.

2.14. Surface Staining of Tumor Cells

The analysis of IFN-γ stimulated tumor cells for the expression of surface markers including CD80, CD86, PDL-1, and HLA-E, along with their respective isotype control antibodies, was performed utilizing flow cytometry. Tumor cells subjected to stimulation with 100 ng/mL IFN-gamma (R&D Systems, Minneapolis, MN, USA) for 72 hours at 37°C in a 5% CO2 incubator were compared to non-stimulated tumor cells. Subsequently, the cells were plated into a 96-well round-bottom plate and centrifuged at 1,200 rpm for 5 minutes, followed by washing with 1x PBS (without CaCl2 & MgCl2). An antibody cocktail, prepared in 1x PBS (without CaCl2 & MgCl2), containing surface staining antibodies at a 1:50 dilution and Fixable Viability dye at a 1:1,000 dilution, was added to the wells. The plate was incubated in the dark at 4°C for 30 minutes. Post-incubation, the cells were washed and fixed using Fluorofix (Biolegend, San Diego, CA, USA) to preserve their state for subsequent flow cytometry analysis. A total of 50,000 cells were collected for comprehensive analyses and evaluation.

2.15. Surface Staining of Immune Cells Including Enriched CD8 T Cells, Total T Cells and NK Cells

The assessment of CTLA-4 expression on various immune cell subsets and NKG2A expression on NK cells was conducted through surface staining methodologies. IL-2 stimulated and non-stimulated peripheral blood mononuclear cells (PBMCs) and specifically enriched CD8$^+$ T cells and

Dual Checkpoint Aptamer Immunotherapy: Unveiling Tailored Cancer Treatment Targeting CTLA-4 and NKG2A

EXPERIMENTAL METHOD DETAILS

NK cells underwent staining using Biotinylated AYA22T aptamers, control aptamers, and a positive control, APC-labeled anti-Human CD152 (CTLA4), PE-labeled anti-Human NKG2A and AF700-labeled anti-Human CD94 surface marker (Biolegend, San Diego, CA, USA). Biotinylated AYA22T aptamers were detected using a subsequent binding with Streptavidin-APC and analyzed via flow cytometry. Post-plating both stimulated and non-stimulated PBMCs, $CD8^+$ T cells and NK cells into 96-well round-bottom plates, centrifugation, was performed followed by washing with 1x PBS (without CaCl2 & MgCl2). Further, a 1:50 dilution of anti-human CTLA-4, anti-Human NKG2A, and anti-Human CD94 antibody or biotinylated AYA22T aptamers followed by Streptavidin-APC and/or Fixable Viability dye was applied to the cells and incubated at 4°C in darkness for 30 minutes. Subsequent steps involved washing and fixation of cells with Fluorofix (Biolegend, San Diego, CA, USA) to preserve cellular integrity for flow cytometry acquisition. A comprehensive analysis was performed on 50,000 collected cells to evaluate the expression levels of CTLA-4 and/or CD94/NKG2A on enriched CD8+ T cells, total T cells, and NK cells, thereby providing crucial insights into the modulation of this immune checkpoint molecule across different cell populations.

2.16. Competitive Binding of AYA22T-R2-13 to CTLA4/NKG2A Surface Receptors on CD8 T cells

The evaluation of AYA22T-R2-13 binding to CTLA-4/NKG2A receptors expressed on activated CD8 T cells was performed using surface staining techniques. Biotinylated AYA22T-R2-13 (5 μM) and bi-control aptamers (5 μM) were separately incubated with or without 5 μM recombinant CTLA4, NKG2A, and CTLA4+NKG2A proteins in 10% FCS cRPMI media at 37◦C for 45 minutes. Subsequently, IL-2-stimulated enriched CD8 T cells underwent staining with a combination of biotinylated(bi)-AYA22T-R2-13, bi-control aptamers, and a negative control (containing only recombinant proteins). This staining process occurred at 4°C in darkness for 30 minutes. The binding of AYA22T-R2-13 to CTLA4 and NKG2A on the CD8 T cells was detected through subsequent binding steps involving Streptavidin-APC and BV510-labeled anti-PD-1, along with Fixable Viability dye. This phase involved incubation at 4°C for 30 minutes. Post-staining, cells were washed, fixed with Fluorofix (Biolegend, San Diego, CA, USA), and analyzed via flow cytometry. A comprehensive analysis was conducted on 50,000 collected cells to assess AYA22T-R2-13 binding to CTLA4/NKG2A and the expression levels of PD-1 on CD8 T cells.

2.17. Detection of Cell Surface CTLA4 binding to AYA22T-R2-13 via Competitive Inhibition Binding Inhibition Assay

The assessment of AYA22T-R2-13 binding to the native cell surface CTLA-4 receptor, expressed on activated CD8 T cells, was conducted through a competitive inhibition binding assay. Enriched IL-2-activated CD8 T cells were incubated with Biotinylated AYA22T-R2-13 or control aptamer (at concentrations of 5, 2, and 1 μM) at 4°C for 45 minutes. Following this incubation, cells were washed and subjected to staining using a cocktail consisting of Streptavidin-PE, APC-labeled anti-human CTLA4, and Fixable Viability dye, and then incubated at 4°C for 30 minutes. The co-staining binding of AYA22T-R2-13 to the CTLA4 receptor on CD8 T cells in the presence of the anti-human

Dual Checkpoint Aptamer Immunotherapy: Unveiling Tailored Cancer Treatment Targeting CTLA-4 and NKG2A

EXPERIMENTAL METHOD DETAILS

CTLA4 antibody was detected using flow cytometry. Subsequently, a comprehensive analysis of 50,000 acquired cells was performed using FlowJo.

2.18. Sulforhodamine B (SRB) Cell Cytotoxicity Assay

Cell viability subsequent to aptamer exposure was assessed using SRB cell cytotoxicity colorimetric assays. Cells at approximately 80% confluence were trypsinized, and around 20,000 tumor cells or PBMCs per well were seeded into a 96-well clear flat-bottom plate and incubated for 24 hours at 37°C with 5% CO2. Different concentrations of aptamers (0 µM, 1 µM, 5 µM, and 10 µM) were prepared and added to their respective wells according to the plate layout. As controls, cell culture medium without cells was used to assess SRB dye binding directly to the wells, while cell culture medium with cells was employed as a control for aptamer influence. Additionally, 1 µL of Doxorubicin from a 20 mM stock was added to a well containing the cells as a positive control, and 1 µL of DMSO was included as a control for Doxorubicin. The plate was then incubated for 72 hours at 37°C with 5% CO2. Post-incubation, cell fixation, staining, solubilization, and spectrophotometric quantification were performed according to the manufacturer’s instructions (Abcam, Waltham, MA, USA).

2.19. CD8+ T-cell Mediated Tumor Cell Killing Assay

The CD8+ T-cell mediated tumor cell killing assay was conducted following a series of controlled steps. Initially, tumor cells underwent stimulation with 100 ng/mL IFN-γ (R&D Systems, Minneapolis, MN, USA) for 72 hours before co-culture with CD8+ T cells. Concurrently, isolated CD8+ T cells were stimulated with 20 ng/mL IL-2 (R&D Systems, Minneapolis, MN, USA) for 24 hours before the co-culture with tumor cells. Subsequently, the stimulated $CD8^+$ T cells were harvested, washed twice with complete RPMI-1640 medium, and quantified. Following quantification, 30 µL of $CD8^+$ T cells per well, accounting for 30,000 cells per well, were plated into a 96-well round-bottom plate. In triplicate, 2 µM CTLA4 aptamers, 10 µg/mL control antibodies, and their respective isotypes were added according to the experimental layout and incubated at 37°C with 346 5% CO2 for 30 minutes. Meanwhile, tumor cells were harvested, washed with complete RPMI medium, and quantified. Subsequently, 100 µL of tumor cells (Target cells) per well with $CD8^+$ T cells (Effector cells), at a 1:1 or 1:3 ratio (T:E) were co-cultured in the wells. Additionally, CD107a, prepared in complete RPMI-1640 medium, was introduced to assess the CD107a expression of $CD8^+$ T cells using flow cytometry at a later stage. All components of the co-culture system were prepared in complete RPMI-1640 medium and incubated for 24 or 48 hours to facilitate the assay. Cytotoxicity was determined by LDH assay.

2.20. NK Cell-mediated Tumor Cell Killing Assay

The NK cell-mediated tumor cell-killing assay involved a methodical series of procedures. Initially, tumor cells were stimulated with 100 ng/mL IFN-gamma (R&D Systems, Minneapolis, MN, USA) for 72 hours prior to co-culture with NK cells. Simultaneously, isolated NK cells were stimulated with 20 ng/mL hIL-2 and 20 ng/mL hIL-15 (R&D Systems, Minneapolis, MN, USA) for 24 hours

EXPERIMENTAL METHOD DETAILS

preceding the co-culture with tumor cells. Subsequent to the stimulation period, the stimulated NK cells were harvested, subjected to two washes with complete RPMI-1640 medium, and enumerated. Following quantification, 30 µL of NK cells per well, with a count of 30,000 cells per well, were plated into a 96-well round-bottom plate. In triplicate, 2 µM CTLA4 aptamers, 10 µg/mL control antibodies, and their corresponding isotypes were added based on the experimental layout and incubated at 37°C in a 5% CO2 incubator for 30 minutes. Meanwhile, tumor cells were harvested, washed with complete RPMI medium, and quantified. Subsequently, 100 µL of tumor cells (Target cells) per well with NK cells (Effector cells), in a 1:1 ratio (T:E) were co-cultured. All components for the co-culture were prepared using complete RPMI-1640 medium and incubated for 48 hours as per the experimental design. Cytotoxicity was determined by LDH assay.

2.21. Killing Assay in the Presence of Human Serum

Human serum was collected from healthy donors. Subsequently, 2 µM of AYA22T-R2-13, AYA22T-R3-25, AYA22T-R2-81, and a control aptamer were either incubated with or without the undiluted human serum (50 µL per well) in a 96-well plate for 15 minutes at 37◦C in a 5% CO2 incubator. Following this incubation, IL-2 stimulated $CD8^+$ T cells (5×10^4) in 100 µL of cRPMI were added to the wells and further incubated for 30 minutes at 37°C in a 5% CO2 incubator. Subsequently, HCT-15 tumor cells (5×10^4) in 150 µL were co-cultured with $CD8^+$ T cells at a Target: effector (T:E) ratio 1:1. The final volume to achieve T:E cell ratio of 250 µL was maintained, and the co-culture was incubated for 48 hours to facilitate the assay. Cytotoxicity was assessed using the LDH assay.

2.22. Lactate Dehydrogenase (LDH) Assay

The assessment of cell viability was conducted utilizing the LDH Assay Cytotoxicity Detection Kit (Roche, Mannheim, Germany). Lactate dehydrogenase (LDH) released from lysed cells served as an indicator of cellular integrity after 48 hours. As a positive control for the LDH assay, 1% Triton X was employed. Following the incubation period, the cell culture plate underwent centrifugation at 500 g for 5 minutes, leading to the collection of the supernatant into a clear 96-well flat-bottom plate. The experimental procedures detailed in the kit protocol were rigorously followed. Subsequently, the colorimetric changes indicative of LDH activity were quantified by spectrophotometric measurements at wavelengths of 490 nm and 620 nm. The percentage cytotoxicity was calculated by the average absorbance values of the triplicate samples and controls, subtracting the background from each, and then substituting the resulting values in the following equation as, % Cytotoxicity = [(Exp. value – Low control) / (High control – Low control)]× 100. Low control represents untreated target cells, and High control represents target cells lysed with 2% Triton X-100 (MilliporeSigma).
