# Supplementary material for: Dual Checkpoint Aptamer Immunotherapy: Unveiling Tailored Cancer Treatment Targeting CTLA-4 and NKG2A
Source: Cancers (Basel). 2024 Mar 4;16(5):1041. doi: 10.3390/cancers16051041 (PMC10931446; doi:10.3390/cancers16051041)
Supplement: Supplementary file 1 [file cancers-16-01041-s001.zip › Supplementary Figures.pdf]

Dual Checkpoint Aptamer Immunotherapy: Unveiling Tailored Cancer Treatment Targeting CTLA-4 and NKG2A

Supplementary Figures

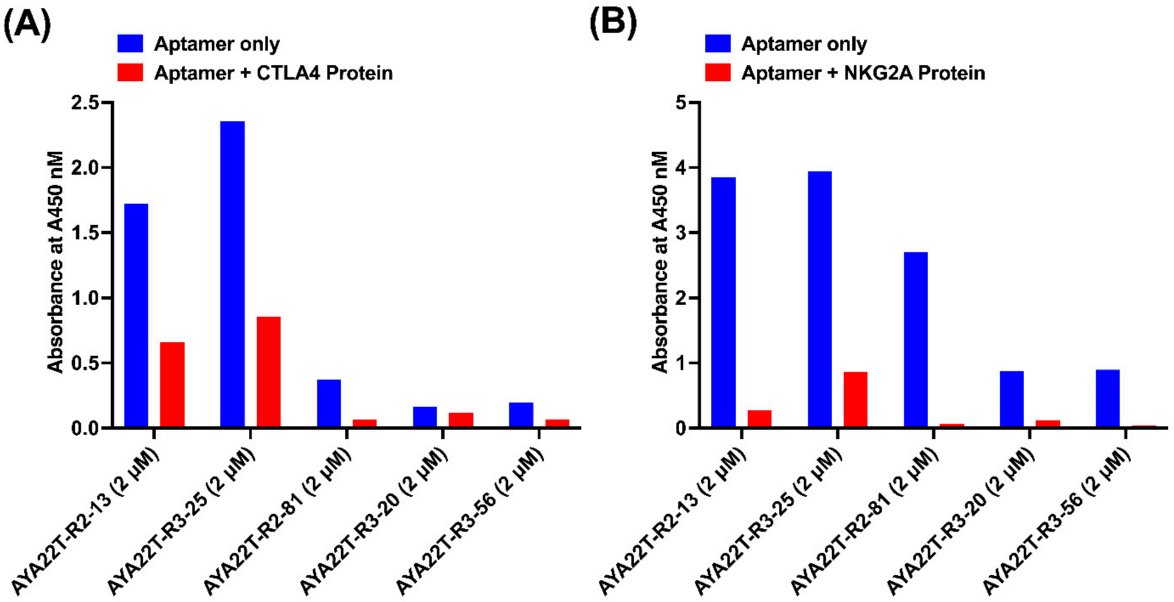

Supplementary Figure S1. Aptamer Binding to Recombinant CTLA4 Protein. An ELISA-based competition assay was conducted to assess the binding of the aptamers to CTLA4 protein. CTLA4 protein at a concentration of 0.26 µM was immobilized on a MaxiSorp plate, and 2 µM biotinylated aptamers were introduced in the absence or presence of 2 µM recombinant CTLA4 (Panel A) or NKG2A (Panel B). The detection of bound aptamers was achieved using streptavidin–HRP. Each recorded value represents the average of duplicate measurements.

Dual Checkpoint Aptamer Immunotherapy: Unveiling Tailored Cancer Treatment Targeting CTLA-4 and NKG2A

Supplementary Figures

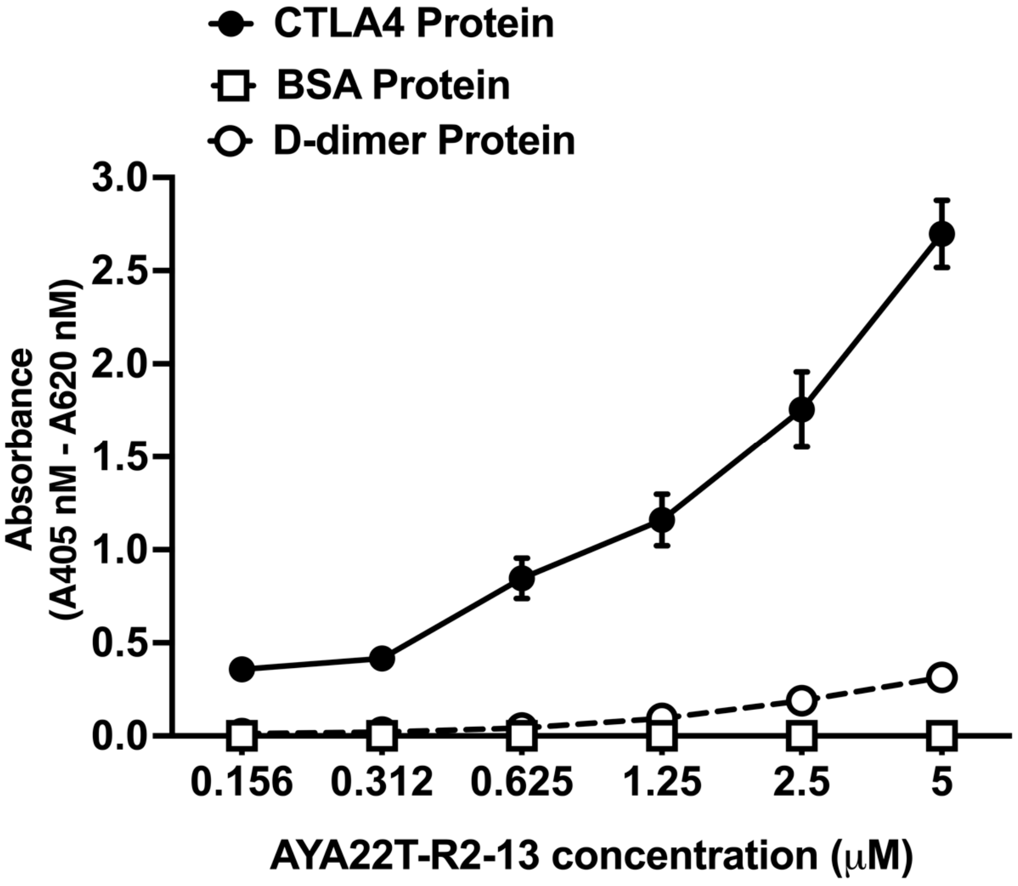

Supplementary Figure S2. Evaluation of AYA22T-R2-13's Specific Binding to CTLA4 Protein. The specific interaction between AYA22T-R2-13 and CTLA4 protein was analyzed through an ELISA assay employing a MaxiSorp plate. The plate was coated with recombinant CTLA4 protein (5 μg/mL), BSA (5 μg/mL), or D-Dimer protein (5 μg/mL). Various concentrations of biotinylated AYA22T-R2-13 were allowed to bind to the immobilized proteins. Detection of the bound aptamers was accomplished using streptavidin–HRP. Each recorded value represents the average of duplicate measurements. Error bars represent mean±SD.

Dual Checkpoint Aptamer Immunotherapy: Unveiling Tailored Cancer Treatment Targeting CTLA-4 and NKG2A

Supplementary Figures

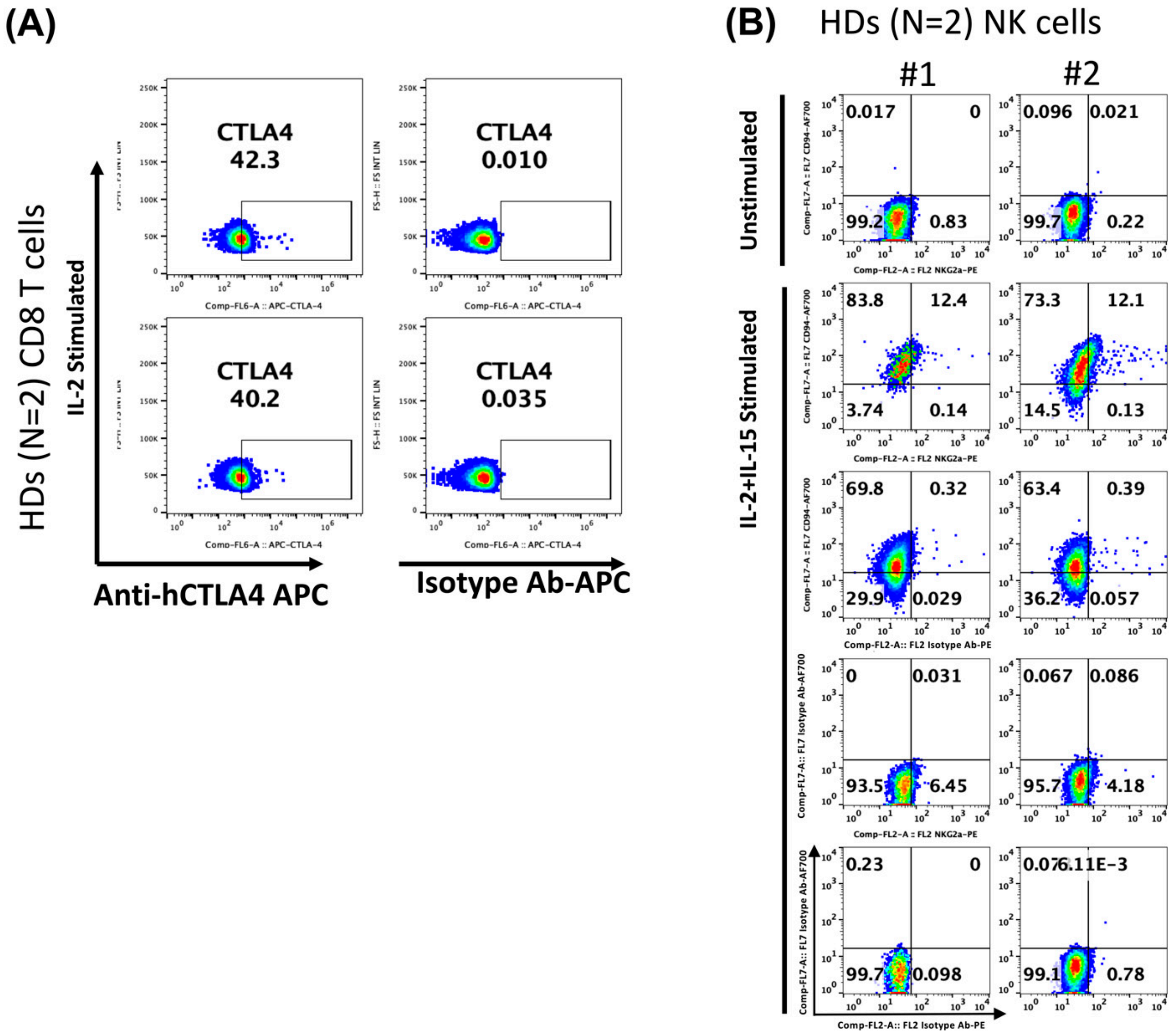

Supplementary Figure S3. CTLA4 expression on activated CD8⁺ T cells. CD8⁺ T cells were enriched using the StemCell negative selection kit and subsequently stimulated with IL-2 (40 ng/ml) for 48 hours. The cells were then subjected to staining using a three-color flow cytometry technique. Fixable Viability Dye eFluor 450 was employed for cell staining, followed by three washes with FACS buffer. Subsequently, cells were fixed by adding 300 ul/well of FluoroFix buffer before analysis. Gating procedures included the initial selection of cells based on forward scatter and side scatter, followed by the selection of singlets and live cells. The Navios EX flow cytometer (Beckman Coulter) was used for the flow cytometry experiments, and FlowJo v10 (FlowJo LLC) was employed to analyze flow cytometry data. All data are representative of at least two independent experiments.

Dual Checkpoint Aptamer Immunotherapy: Unveiling Tailored Cancer Treatment Targeting CTLA-4 and NKG2A

Supplementary Figures

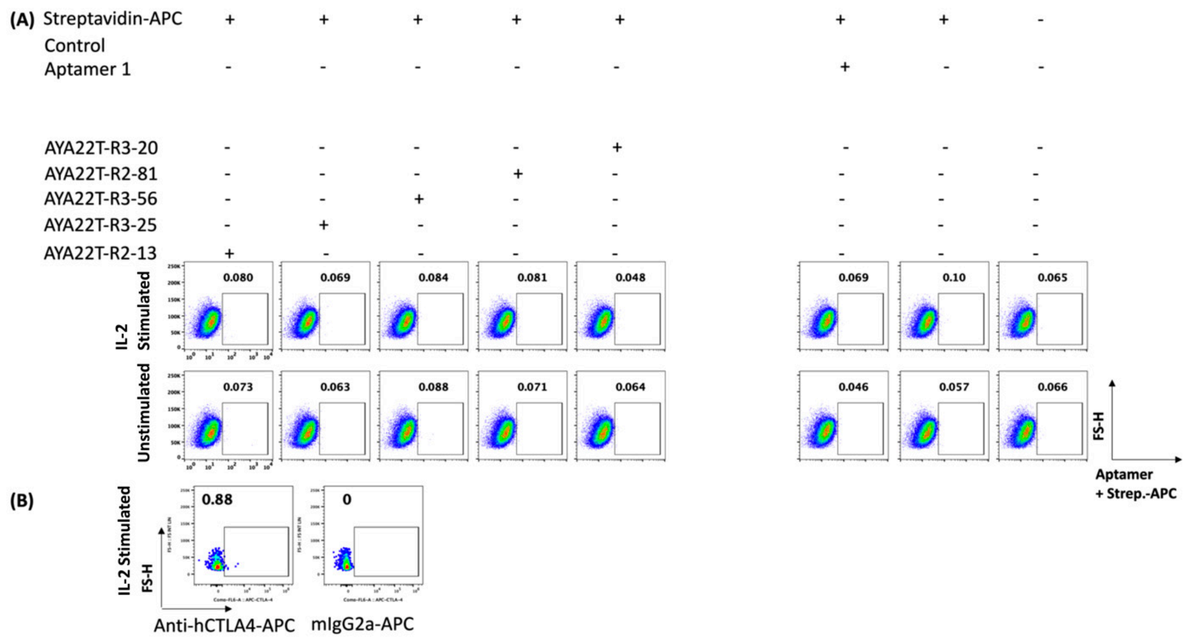

Supplementary Figure S4. Detection of Non-Specific Binding of AYA22T-Aptamers on the Cell Surface. 293T cells were utilized to assess the nonspecific binding of AYA22T-aptamers. The cells were stimulated with or without IL-2 (40 ng/ml) for 48 hours. Initially, cells were incubated with biotinylated AYA22T-aptamers and control aptamer for 30 minutes at 4°C, followed by washing. Subsequently, cells underwent staining using a two-color flow cytometry technique. Fixable Viability Dye eFluor 450 was included, along with SA-APC or anti-human APC-labeled CTLA4 antibody and its isotype control antibody for cell staining. This was followed by three washes with FACS buffer. Cells were then fixed by adding 300 ul/well of FluoroFix buffer before analysis. Gating procedures included the initial selection of cells based on forward scatter and side scatter, followed by the selection of singlets and live cells. The Navios EX flow cytometer (Beckman Coulter) was employed for the flow cytometry experiments, and FlowJo v10 (FlowJo LLC) was used for the analysis of flow cytometry data. All data are representative of at least two independent experiments.

Dual Checkpoint Aptamer Immunotherapy: Unveiling Tailored Cancer Treatment Targeting CTLA-4 and NKG2A

Supplementary Figures

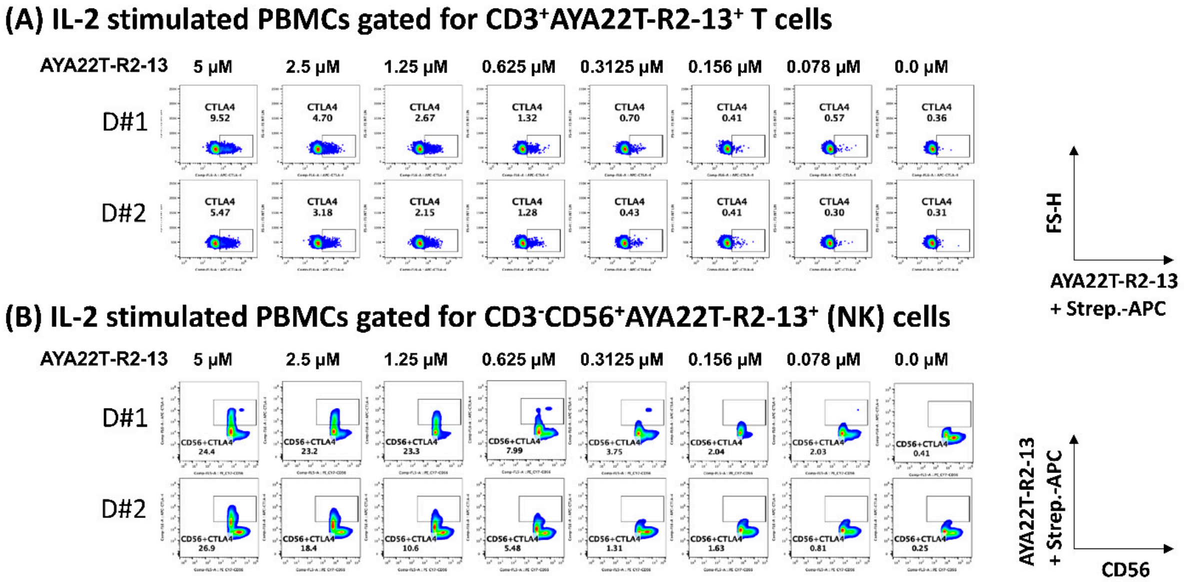

Supplementary Figure S5. Binding of AYA22T-R2-13 to the cell surface of T cells and NK cells. (A) Assessment of AYA22T-R2-13 binding to $CD3^+$ T cells and (B) $CD56^+CD3^-$ (NK) cells from IL-2 (20 ng/ml) stimulated human PBMCs from healthy donors was determined. Cells were incubated first with serially diluted biotinylated AYA22T-R2-13 for 30 minutes at 4°C, followed by washing and staining with anti-human CD3, anti-human CD56, and SA-APC antibodies (added at a 1:50 dilution in FACS buffer), including Fixable Viability Dye eFluor 450 for 30 minutes at 4°C. This was followed by three washes with FACS buffer. Subsequently, cells were fixed by adding 300 ul/well of FluoroFix buffer before analysis. Gating was performed by initially selecting cells based on forward scatter and side scatter, followed by the selection of singlets and live cells. The Navios EX flow cytometer (Beckman Coulter) was used for the flow cytometry experiments, and FlowJo v10.8.1 (Becton Dickinson Life Sciences) was employed for the analysis of flow cytometry data. All data are representative of at least three independent experiments.

Dual Checkpoint Aptamer Immunotherapy: Unveiling Tailored Cancer Treatment Targeting CTLA-4 and NKG2A

Supplementary Figures

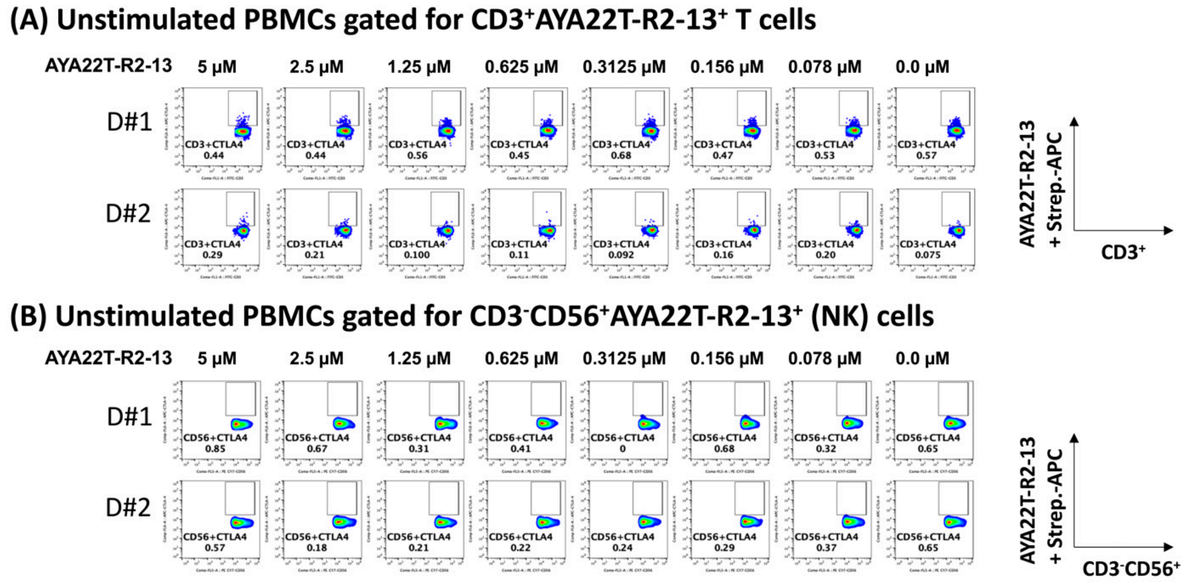

Supplementary Figure S6. Binding of AYA22T-R2-13 to the cell surface of T cells and NK cells in human PBMCs. (A) Assessment of AYA22T-R2-13 binding to $CD3^+$ T cells and (B) $CD56^+CD3^-$ (NK) cells from unstimulated human PBMCs from healthy donors was determined. Cells were incubated first with serially diluted biotinylated AYA22T-R2-13 for 30 minutes at 4°C, followed by washing and staining with anti-human CD3, anti-human CD56, and SA-APC antibodies (added at a 1:50 dilution in FACS buffer), including Fixable Viability Dye eFluor 450 for 30 minutes at 4°C. This was followed by three washes with FACS buffer. Subsequently, cells were fixed by adding 300 ul/well of FluoroFix buffer before analysis. Gating was performed by initially selecting cells based on forward scatter and side scatter, followed by the selection of singlets and live cells. The Navios EX flow cytometer (Beckman Coulter) was used for the flow cytometry experiments, and FlowJo v10.8.1 (Becton Dickinson Life Sciences) was employed to analyze flow cytometry data. All data are representative of at least three independent experiments.

Dual Checkpoint Aptamer Immunotherapy: Unveiling Tailored Cancer Treatment Targeting CTLA-4 and NKG2A

Supplementary Figures

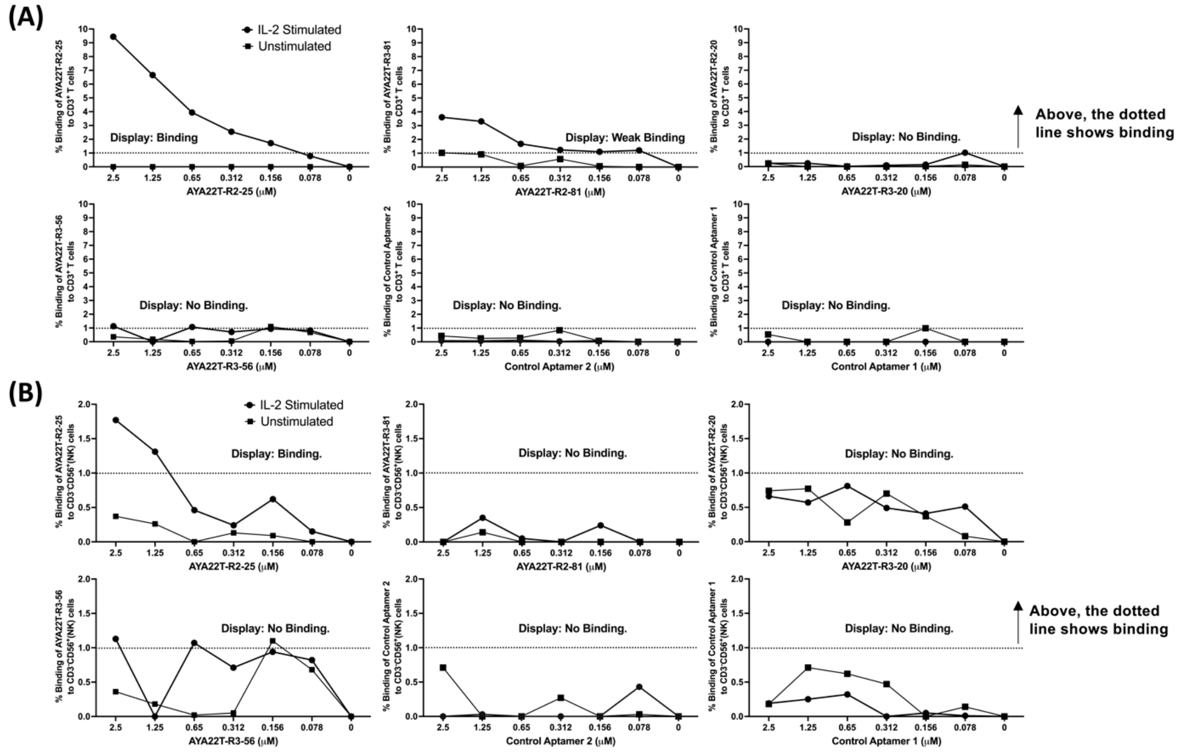

Supplementary Figure S7. Binding of AYA22T aptamers to the cell surface of T cells and NK cells in human PBMCs. (A) Assessment of AYA22T-R2-25, AYA22T-R3-81, AYA22T-R2-20, AYA22T-R3-56 binding to $CD3^+$ T cells and **(B)** Assessment of AYA22T-R2-25, AYA22T-R3-81, AYA22T-R2-20, AYA22T-R3-56 binding to $CD56^+CD3^-$ (NK) cells in IL-2-stimulated versus unstimulated human PBMCs from healthy donors. Cells were incubated first with serially diluted biotinylated AYA22T aptamers and control aptamers for 30 minutes at 4°C, followed by washing and staining with anti-human CD3, anti-human CD56, and SA-APC antibodies (added at a 1:50 dilution in FACS buffer), including Fixable Viability Dye eFluor 450, for 30 minutes at 4°C. This was followed by three washes with FACS buffer. Subsequently, cells were fixed by adding 300 ul/well of FluoroFix buffer before analysis. Gating was performed by initially selecting cells based on forward scatter and side scatter, followed by the selection of singlets and live cells. The Navios EX flow cytometer (Beckman Coulter) was used for the flow cytometry experiments, and FlowJo v10.8.1 (Becton Dickinson Life Sciences) was employed to analyze flow cytometry data. All data are representative of at least two independent experiments.

Dual Checkpoint Aptamer Immunotherapy: Unveiling Tailored Cancer Treatment Targeting CTLA-4 and NKG2A

Supplementary Figures

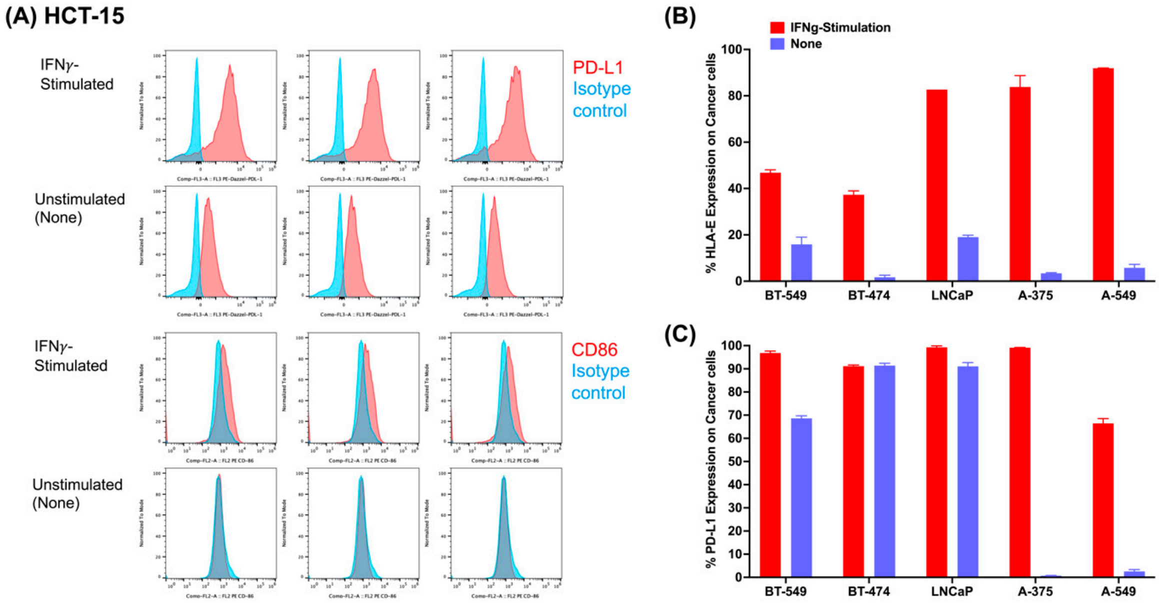

Supplementary Figure S8. Expression of CD86, HLA-E, and PD-L1 by various human tumor cell lines with or without IFN-γ stimulation. Human tumor cell lines, including HCT-15, BT-549, BT-474, LNCaP, A-375, and A-549, were cultured in their respective media until cells reached approximately 80% confluency. Subsequently, cells were stimulated with or without 100 ng/mL of IFN-γ and incubated for 24 hours at 37°C with 5% CO2. After incubation, cells were trypsinized, washed, and stained with anti-PD-L1, anti-CD86, and anti-HLA-E antibodies at a 1:50 dilution in FACS buffer, including Fixable Viability Dye eFluor 450, for 30 minutes at 4°C. This was followed by three washes with FACS buffer. Subsequently, cells were fixed by adding 300 ul/well of FluoroFix buffer before analysis. Gating was performed by initially selecting cells based on forward scatter and side scatter, followed by the selection of singlets and live cells. The Navios EX flow cytometer (Beckman Coulter) was used for the flow cytometry experiments, and FlowJo v10.8.1 (Becton Dickinson Life Sciences) was employed to analyze flow cytometry data. Data are represented as MFI shift on stimulated versus unstimulated HCT-15 **(A)** and quantified as MFI (mean fluorescence intensity) in bar graph format for BT-549, BT-474, LNCaP, A-375, and A-549 **(B-C)**. Each recorded value represents the average of duplicate measurements. Error bars represent mean±SD.

Dual Checkpoint Aptamer Immunotherapy: Unveiling Tailored Cancer Treatment Targeting CTLA-4 and NKG2A

Supplementary Figures

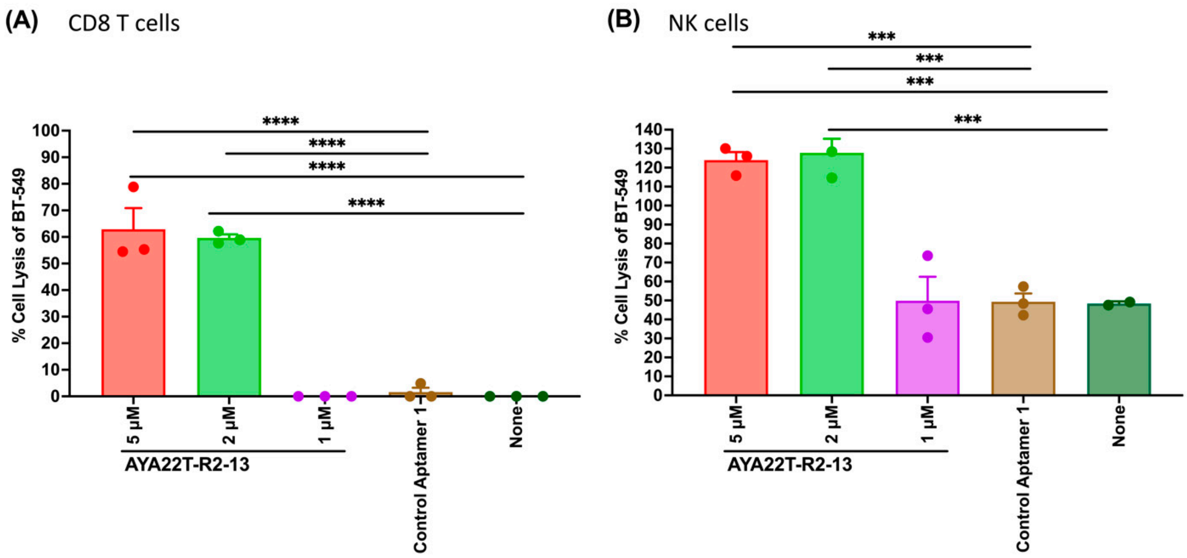

Supplementary Figure S9. **Titration of AYA22T-R2-13 dosing concentration for in vitro tumor cell lysis by CD8 T cells and NK cells.** Isolated CD8 T cells and NK cells from healthy donors' PBMCs were incubated with recombinant hIL-2 (20 ng/ml) for 24 hours. Subsequently, CD8 T cells and NK cells, respectively, were cocultured with hIFN-γ-stimulated tumor cells BT-549 at a target/effector cell ratio of 1:3 (T:E) in the presence or absence of CTLA4/NKG2A aptamer (AYA22T-R2-13), along with or without a control aptamer (negative controls), for 48 hours. Percent cell lysis/killing was assessed by lactate dehydrogenase (LDH) assay. The error bars depict the mean ± SD of CD8 T cells and NK cells from a total of N=3 healthy donors, each contributing to an independent experimental set. *** denotes $p < 0.001$, and **** denotes $p < 0.0001$.

Dual Checkpoint Aptamer Immunotherapy: Unveiling Tailored Cancer Treatment Targeting CTLA-4 and NKG2A

Supplementary Figures

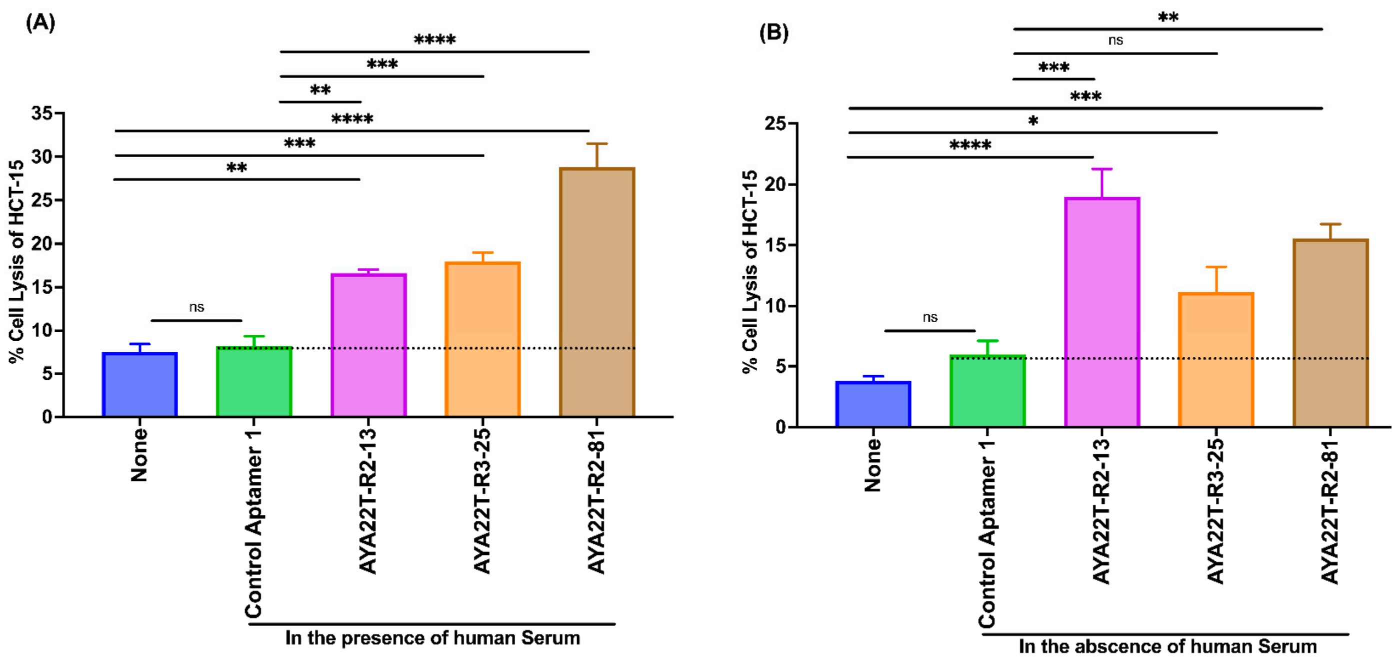

Supplementary Figure S10. Tumor cell lysis activity of AYA22T aptamers by CD8 T cells remains uncompromised in the presence of human serum. Isolated CD8 T cells from healthy donors' PBMCs were incubated with recombinant hIL-2 (20 ng/ml) for 24 hours. CD8 T cells were then incubated in the presence or absence of CTLA4/NKG2A aptamers (AYA22T-R2-13, AYA22T-R3-25, and AYA22T-R2-81), along with a control aptamer (negative control), at a concentration of 2 uM. This incubation was carried out in the presence or absence of undiluted human serum for 30 minutes at 37°C. Subsequently, CD8 T cells were cocultured with hIFN-γ-stimulated tumor cells (HCT-15) at a target/effector cell ratio of 1:1 (T:E) for 48 hours at 37°C in a 5% CO2 incubator. Percent cell lysis/killing was assessed by lactate dehydrogenase (LDH) assay. The error bars depict the mean ± SD of CD8 T cells from a total of N=3 healthy donors, each contributing to an independent experimental set. * denotes $p < 0.05$, ** denotes $p < 0.01$, *** denotes $p < 0.001$, and **** denotes $p < 0.0001$.

Dual Checkpoint Aptamer Immunotherapy: Unveiling Tailored Cancer Treatment Targeting CTLA-4 and NKG2A

Supplementary Figures

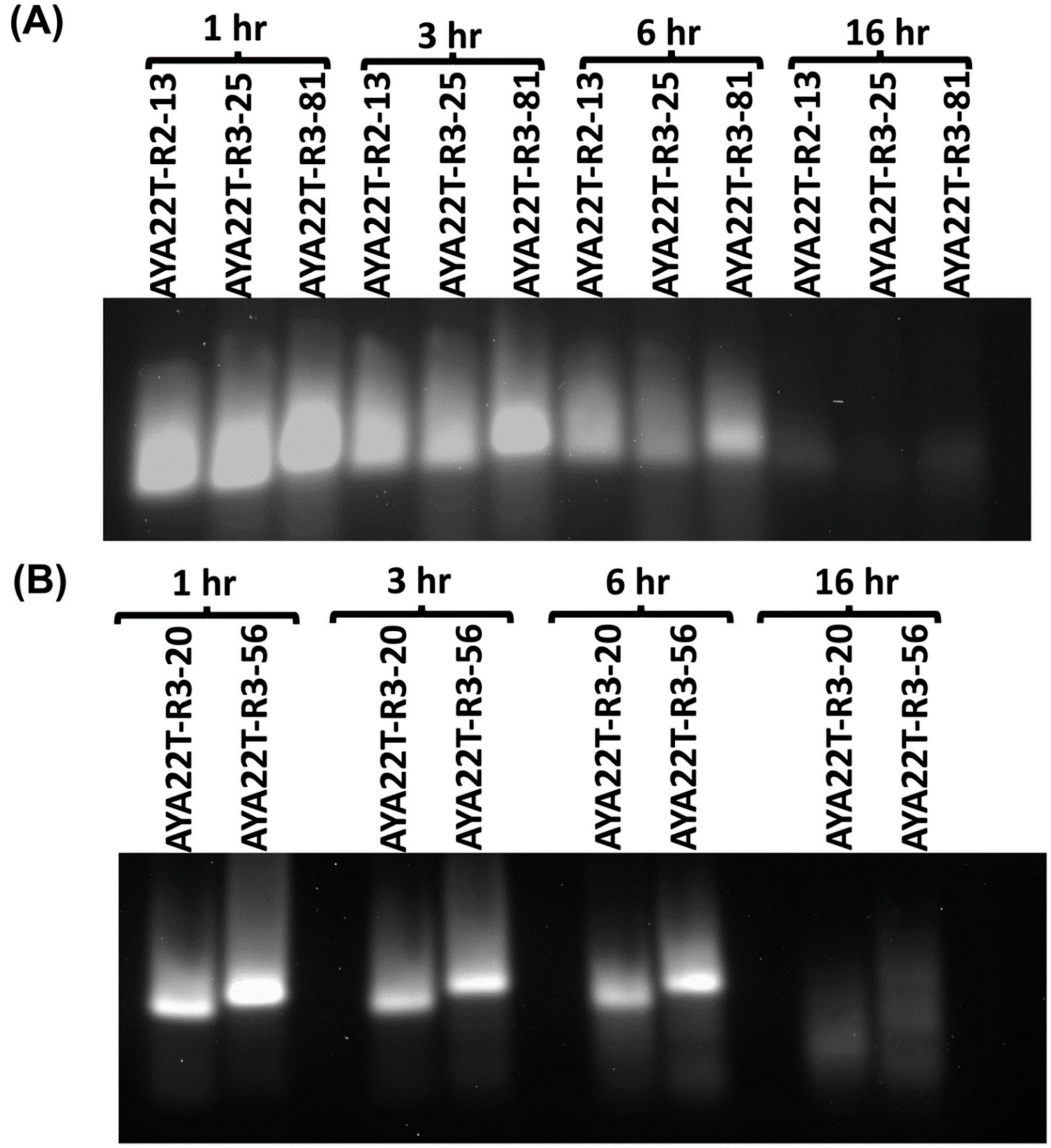

Supplementary Figure S11. CTLA4/NKG2A aptamers (AYA22T) exhibit relative stability in human serum. Gel electrophoresis revealed that AYA22T aptamers in human serum, during the incubation periods of 1, 3, 6, and 16 hours at 37°C, remained nearly intact after 6 hours and showed signs of degradation at the 16-hour incubation period. All data are representative of at least two independent experiments.

Dual Checkpoint Aptamer Immunotherapy: Unveiling Tailored Cancer Treatment Targeting CTLA-4 and NKG2A

Supplementary Figures

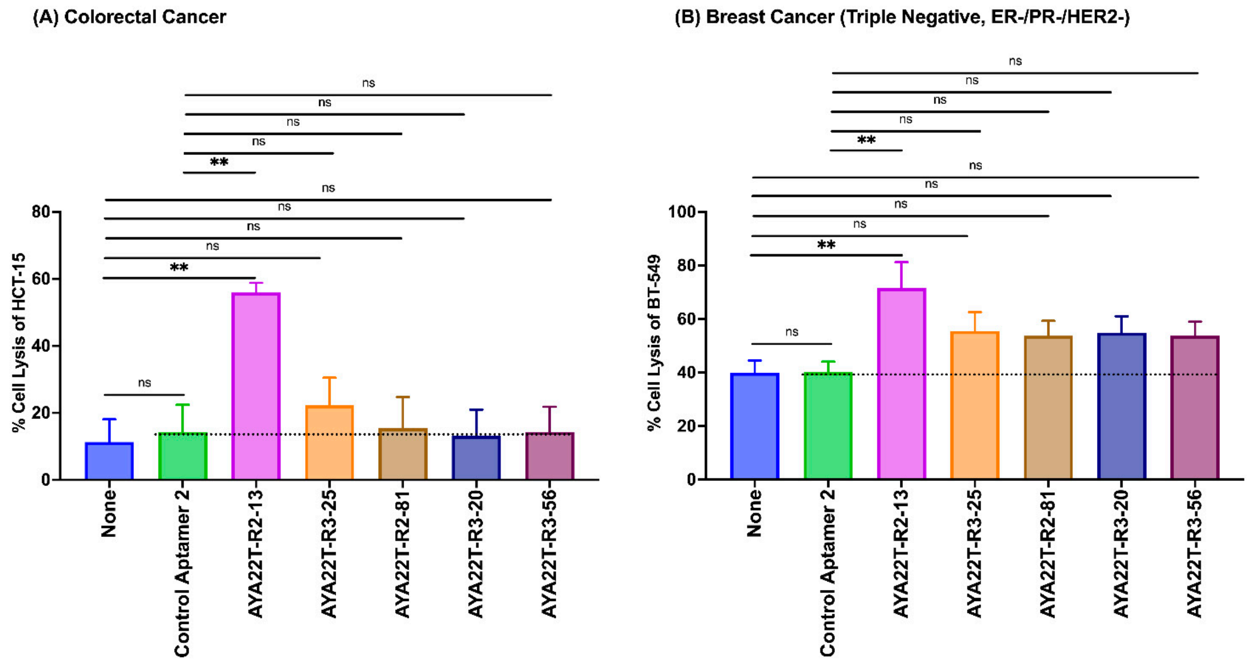

Supplementary Figure S12. AYA22T-R2-13 enhances NK cell cytolytic activity in vitro. NK cells isolated from healthy donors' PBMCs were incubated with recombinant hIL-2 (20 ng/ml) for 24 hours. Subsequently, NK cells were cocultured with hIFN-γ-stimulated tumor cells, HCT-15 and BT-549, at a target/effector cell ratio of 1:3 (T:E). This coculture was performed in the presence or absence of CTLA4/NKG2A aptamers (AYA22T-R2-13, AYA22T-R3-25, AYA22T-R2-81, AYA22T-R3-20, and AYA22T-R3-56), along with or without a control aptamer (negative controls), for 48 hours at 37°C in a 5% CO2 incubator. Percent cell lysis/killing was assessed using the lactate dehydrogenase (LDH) assay. The error bars depict the mean ± SD of NK cells from a total of N=3 healthy donors, each contributing to an independent experimental set. ** denotes $p < 0.01$.
