# Supplementary material for: Dual Checkpoint Aptamer Immunotherapy: Unveiling Tailored Cancer Treatment Targeting CTLA-4 and NKG2A
Source: Cancers (Basel). 2024 Mar 4;16(5):1041. doi: 10.3390/cancers16051041 (PMC10931446; doi:10.3390/cancers16051041)
Supplement: Supplementary file 1 [file cancers-16-01041-s001.zip › Table S1.pdf]

Supplementary Table S1: Sequences of CTLA4 aptamers

Aptamer Name	Sequence
AYA22T-R2-13	ACACdUdUdUdUCCCCCACCdUGAdUCCdUCAGdUdUCCGGAAAAGdUGdU
AYA22T-R2-81	ACdUdUACAACdUGCGCACGCGGGACCdUCCGAGAGdUdUGdUAAGdU
AYA22T-R2-63	dUGAdUdUdUAACdUdUCdUAGAGGGGGGGGdUGGGGdUAGdUdUAAAdUCA
AYA22T-R3-56	dUdUGdUdUGCAGGCGACGdUGCGdUGGGCGCACGGCCdUGCAACAA
AYA22T-R3-20	GACCdUGCCGGCGGAGCdUGCGdUCCCGCdUGCACCGGCAGGdUC
AYA22T-R3-25	GACdUdUCGCGGAGGdUAAACdUGCACGGGGGdUGCCGCGAAGdUC
