# Supplementary material for: Dual Checkpoint Aptamer Immunotherapy: Unveiling Tailored Cancer Treatment Targeting CTLA-4 and NKG2A
Source: Cancers (Basel). 2024 Mar 4;16(5):1041. doi: 10.3390/cancers16051041 (PMC10931446; doi:10.3390/cancers16051041)
Supplement: Supplementary file 1 [file cancers-16-01041-s001.zip › Table S2.pdf]

Supplementary Table S2: Key Resources of Materials and Methods

Reagent/Resource	Source	Identifier	Volume
Antibodies			
Anti-Human CD3-Alexa Fluor700 (Clone: OKT3)	Biolegend	317340	0.1µg
Anti-Human CD3-FITC, Clone: (Clone: OKT3	Biolegend	317306	0.2 µg
Anti-Human CD107a (LAMP-1)-PE/Cyanine7 (Clone: H4A3)	Biolegend	328618	0.2 µg
Anti-Human Perforin-APC (Clone: B-D48)	Biolegend	353312	0.012 µg
Anti-Human/mouse Granzyme B FITC (Clone: QA18A28)	Biolegend	396404	0.025 µg
Anti-human CD80 BV510 (Clone: 2D10)	Biolegend	305234	0.16 µg
Anti-human CD86 PE (Clone: BU63)	Biolegend	374206	0.05 µg
Anti-human CD274 (B7-H1), PDL-1 PE/Dazzle™594 (Clone: 29E.2.A3)	Biolegend	329732	0.2 µg
Anti-Human HLA-E-Alexa Fluor647 (Clone: 3D12/HLA-E)	BD Biosciences	567418	0.5 µg
Anti-Human CD56 (NCAM) PE Cyanine7 (Clone: QA17A16)	Biolegend	392412	0.025 µg
Anti-Human CD152 (CTLA4) APC (Clone: BN13)	Biolegend	369612	0.1µg
Anti-Human NKG2A PE (Clone: 131411)	R&D Systems	FAB1059P	0.025 µg
Anti-Human CD94 Alexa Fluor® 700-conjugated Antibody 131412	R&D Systems	FAB1058N	0.2 µg
Streptavidin-APC	Biolegend	405243	0.50 µg
Anti-Human CTLA-4(Ipilimumab Biosimilar) (Clone: Ipilimumab)	InVivoSIM	SIM0004	7.37µg
Human IgG1 isotype control	InVivoPlus	BP0297	7.89µg

Anti-Human CTLA-4(CD152) (Clone: BN13)	InVivoMAb	BEO190	7.27µg
Mouse IgG2a isotype control (Clone: C1.18.4)	InVivoMAb	BE0085	9.24µg
Mouse IgG1, k isotype PE (Clone: MOPC-21)	Biolegend	400112	0.2 µg
Mouse IgG1, k isotype BV510 (Clone: MOPC-21)	Biolegend	400171	0.1 µg
Mouse IgG1, k isotype APC (Clone: MOPC-21)	Biolegend	400120	0.2 µg
Mouse IgG1, k isotype PE/Cyanine7 (Clone: MOPC-21)	Biolegend	400126	0.2 µg
Mouse IgG2a, k isotype APC (Clone: MOPC-21)	Biolegend	400220	0.2 µg
Rat IgG1, k isotype AF488 (Clone: MOPC-21)	Invitrogen	53-4301-80	0.2 µg
Fixable Viability Dye eFluor™ 450	Invitrogen	65-0863-14	1:1000
Cell staining buffer	BioLegend,	B330711	
FluroFix™ Buffer	BioLegend	B331501	
BD Cytofix/Cytoperm Fixation and Permeabilization Kit	BD Biosciences	554714	
**Cell Lines**			
HCT-15; Colorectal Carcinoma; Human	ATCC	CCL-225	
COLO-205; Colon Adenocarcinoma; Human	ATCC	CCL-222	
LNCaP clone FGC; Prostate carcinoma; Human	ATCC	CRL1740	
BT-549; Breast Ductal Carcinoma; Human	ATCC	HTB-122	
A-375; Melanoma; Human	ATCC	CRL-1619	

A-549; Lung Carcinoma; Human	ATCC	CCL-185	
BT-474; Breast Ductal Carcinoma; Human	ATCC	HTB-20	
**Media + other supplements**			
RPMI-1640, high glucose with L-Glutamine, with HEPES	ATCC	30-2001	
RPMI-1640	Gibco	11875093	
Dulbecco's Modified Eagle Medium (DMEM) (1X)	Gibco	11995-065	
F-12K Kaighn's Modification of Ham's F-12 with L-Glutamine	ATCC	30-2004	
Hybri-Care Medium (powder) with L-Glutamine, with HEPES	ATCC	46-X™	
HyClone Standard FBS, US Origin, 500 mL, Heat-inactivated	Cytiva life Sciences	SH30088.03HI	10%
HEPES (1M)	Gibco	15630-080	25mM
MEM Non-essential Amino Acids Solution (100x)	Gibco	11140-050	1X
Sodium Pyruvate (100mM)	Gibco	11360-070	1mM
Pen Strep (Penicillin Streptomycin) 10,000 U/mL – Penicillin + 10,000 µg/mL Streptomycin	Gibco	15-140-122	100 U/mL Penicillin 100 µg/mL Streptomycin
Sodium bicarbonate	Sigma-Aldrich	S6014-1KG	
StemPro™ Accutase™ Cell Dissociation Reagent	Gibco	A11105-01	
0.25% Trypsin 2.21 mM EDTA, 1x (-) sodium bicarbonate, sterile	Corning	25-053-CI	
**Other Requirements**			

Buffy coat	Carter Blood Care, Bedford Texas		
Ficoll-Paque PLUS density gradient media	Cytiva life Sciences	17144002	
PBS pH 7.4 (1X)	Gibco	10010-031	
Ultra-Pure 0.5 M EDTA, pH 8.0	Invitrogen	15575-038	
DMSO (Dimethyl Sulfoxide)	Corning	25-950-CQC	
EasySep^{TM} Human CD8+ T cell Isolation Kit	Stemcell Technologies	17953	
EasySep™ Human NK Cell Enrichment Kit	Stemcell Technologies	19055	
Recombinant human IFN-gamma, E. coli derived, 100 ug	R&D Systems	285-IF/CF	100 ng/mL
Recombinant human IL-2, E. coli derived, 50 ug	R&D Systems	202-IL/CF	20 ng/mL
Recombinant Human IL-15, E. coli derived, 10 ug	R&D Systems	BT-015/CF	20 ng/mL
SPHERO™ Streptavidin Coated Flow Cytometry Multiplex Bead Assay Particles	Spherotech	SVFB-2558-6K	
Gator™ Streptavidin (SA) Probes	Gator Bio	160002	
Cytotoxicity Detection Kit (LDH)	Roche Diagnostics	11644793001	
SRB Assay/Sulforhodamine B Assay	Abcam	Ab235935	
Human CTLA-4/CD152 Protein, His Tag, active dimer	Acro Biosystems	CT4-H52H9	
Human NKG2A/CD159a Protein, His Tag	Acro Biosystems	NKA-H5244	
Pierce High Sensitivity Streptavidin-HRP	Thermo Fisher Scientific	21130	

1-Step Ultra TMB-ELISA	Thermo Fisher Scientific	34028	
20X PBS Tween™-20	Thermo Fisher Scientific	28352	
Magnesium chloride solution 1M	Sigma-Aldrich	M1028	
Sulfuric Acid Solution 6N	Millipore	SX1243M-6	
BupH™ Carbonate-Bicarbonate Buffer	Thermo Fisher Scientific	28382	
**Software**			
RNAcomposer tool	RNAComposer ver. 1.0	https://rnacomposer.cs.put.poznan.pl	
HADDOCK 2.4	Bonvinlab	https://wenmr.science.uu.nl/haddock2.4/	
PyMOL	Schrodinger	https://pymol.org/2/	
FlowJo™ v10.8.1	BD Biosciences	https://www.flowjo.com/	
Prism 9.4.0	GraphPad Software	https://www.graphpad.com/scientific-software/prism/	
**Instruments**			
Navios EX System, 10 colors/3 lasers (5+3+2 configuration)	Beckman Coulter	B83535	
BioTek Synergy HTX Multimode Reader	Agilent Technologies		
